# Exploring China’s Clean Air Act and associated cardiovascular disease risk: a prospective, quasi-experimental, and causal inference modelling study

**DOI:** 10.1016/j.lanplh.2026.101513

**Published:** 2026-09-01

**Authors:** Dong Sun, Xia Meng, Jingcen Hu, Canqing Yu, Dianjianyi Sun, Yuanjie Pang, Pei Pei, Ling Yang, Yiping Chen, Huaidong Du, Xiaofang Chen, Xiaoming Yang, Junshi Chen, Zhengming Chen, Liming Li, Haidong Kan, Jun Lv, China Kadoorie Biobank Collaborative Group, Junshi Chen, Zhengming Chen, Robert Clarke, Rory Collins, Liming Li, Jun Lv, Richard Peto, Robin Walters, Daniel Avery, Maxim Barnard, Derrick Bennett, Ruth Boxall, Ka Hung Chan, Yiping Chen, Zhengming Chen, Charlotte Clarke, Johnathan Clarke, Robert Clarke, Huaidong Du, Ahmed Edris Mohamed, Hannah Fry, Simon Gilbert, Pek Kei Im, Andri Iona, Maria Kakkoura, Christiana Kartsonaki, Kshitij Kolhe, Hubert Lam, Kuang Lin, James Liu, Mohsen Mazidi, Iona Millwood, Sam Morris, Qunhua Nie, Alfred Pozarickij, Maryam Rahmati, Paul Ryder, Dan Schmidt, Becky Stevens, Iain Turnbull, Robin Walters, Baihan Wang, Lin Wang, Neil Wright, Ling Yang, Xiaoming Yang, Pang Yao, Jun Lv, Canqing Yu, Dianjianyi Sun, Yuanjie Pang, Can Hou, Qingmei Xia, Chao Liu, Pei Pei, Lang Pan, Xiao Han, Honglu Bian, Xinxin Chen, Zengchang Pang, Ruqin Gao, Shanpeng Li, Haiping Duan, Shaojie Wang, Yongmei Liu, Ranran Du, Liang Cheng, Xiaocao Tian, Hua Zhang, Dan Hu, Xiaoyan Zheng, Yujie Wang, Wei Sun, Shichun Yan, Xiaoming Cui, Chi Wang, Zhenyuan Wu, Lishun Zhai, Zhaoxi Pang, Shiwen Dong, Huiming Luo, Jinyan Chen, Bin He, Dingwei Sun, Xingren Wang, Tingting Ou, Xiangyang Zheng, Dewei Zheng, Shuai Yang, Yilei Li, Lihui Li, Xingjiao Chen, Jinyi Zhou, Ran Tao, Jian Su, Xikang Fan, Zongming Cheng, Yuxiao Huang, Yan Lu, Yujie Hua, Li Xing, Shuxian Wang, Jianrong Jin, Juping Ma, Jinchao Liu, Kaifei Zhu, Hongfu Ren, Xingfeng Shen, Ge Zhong, Wei Mao, Zhenzhen Lu, Ling He, Lifang Zhou, Changping Xie, Jian Lan, Tingping Zhu, Jinxue Tan, Liuping Wei, Liyuan Zhou, Sisi Wang, Xianping Wu, Ningmei Zhang, Xiaofang Chen, Xiaoyu Chang, Zhuo Wang, Yujin He, Mingqiang Yuan, Xia Wu, Xiaofang Chen, Zhaodong Wang, Qiang Sun, Yang Lin, Faqing Chen, Xiaolan Ren, Lijun Chang, Feiming Zhong, Jianjun Feng, Weijie Hu, Xiaofang Zhang, Yalin Chen, Fei Wang, Jun Wang, Linqi Diao, Wanshen Guo, Zhiwei Han, Dongyang Zhao, Dengjun Zhu, Kai Kang, Shixian Feng, Huizi Tian, Yali Yan, Bing Han, Li Gao, Shaofang Li, Huafei Feng, Wei Tang, Xiaolin Li, Huarong Sun, Xiaocong Zhao, Ying Li, Chen Hu, Pan He, Xukui Zhang, Yuanyuan Jin, Hesheng Zhang, Min Yu, Ruying Hu, Hao Wang, Weiwei Gong, Jieming Zhong, Meng Wang, Chunxiao Xu, Keqing Gong, Hao Xu, Yuan Cao, Kaixu Xie, Lingli Chen, Xiaomei Tu, Chen Chen, Xiaojun Li, Li Yin, Huilin Liu, Yuan Liu, Yi Liu, Lei Yin, Xian Xie, Jing Wang, Bo Xiao, Pingsheng Lou, Yuan Peng, Libo Zhang, Chan Qu, Qili Jiang, Yanling Chen, Yan Zhao

**Affiliations:** a Department of Epidemiology & Biostatistics, School of Public Health, Peking University, Beijing, China; b School of Public Health, Key Laboratory of Public Health Safety of the Ministry of Education, National Health Commission Key Laboratory of Health Technology Assessment, Integrated Research on Disaster Risk, International Centers of Excellence on Risk Interconnectivity and Governance on Weather/Climate Extremes Impact and Public Health, Fudan University, Shanghai, China; c Peking University Center for Public Health and Epidemic Preparedness & Response, Beijing, China; d Key Laboratory of Epidemiology of Major Diseases, Peking University, Ministry of Education, Beijing, China; e Clinical Trial Service Unit and Epidemiological Studies Unit, Nuffield Department of Population Health, University of Oxford, Oxford, United Kingdom; f Chengdu Medical College, Chengdu, China; g China National Center for Food Safety Risk Assessment, Beijing, China; h State Key Laboratory of Vascular Homeostasis and Remodeling, Peking University, Beijing, China

## Abstract

**Background:**

Substantial improvements in air quality have been recorded following the implementation of China's Clean Air Act (CCAA) in 2013. However, the association between CCAA implementation and individual-level cardiovascular disease (CVD) risk remains unclear. We aimed to examine the long-term association between CCAA implementation and individual-level predicted CVD risk.

**Methods:**

In this prospective, quasi-experimental study, we used data from the China Kadoorie Biobank, a prospective cohort study that recruited participants from five urban and five rural areas across China between 2004 and 2008, with three resurveys conducted after the baseline survey (in 2008, 2013–14, and 2020–21). We included 34 862 individuals (mean age 51·3 years) who participated in at least one resurvey and had no history of CVD at baseline. Participants were classified into intervention (n=25 497) and control (n=9365) groups based on the local government’s targets for particulate matter reduction. We estimated the 10-year risk of incident CVD morbidity or mortality using a validated risk prediction model. We used a difference-in-difference model to assess the long-term association between CCAA implementation and predicted risk, with adjustments made for regional confounders and individual-level characteristics, including demographics, lifestyle factors, medical history, and indoor air pollution exposure. The relationship between changes in long-term exposure to PM_2·5_, PM_10_, and O_3_ and predicted risk after CCAA implementation was analysed using a linear model. The estimated risk differences associated with air pollutant changes were estimated based on the magnitude of changes and their corresponding effect sizes.

**Findings:**

After the CCAA was implemented, PM_2·5_ and PM_10_ concentrations declined in both groups, but O_3_ concentrations increased. The intervention group showed a 3·95% (95% CI 3·18–4·72%) lower increase in predicted risk than the control group, with larger estimated differences under stricter enforcement. Between 2013 and 2021, each 10 μg/m^3^ change in PM_2·5_ concentration was positively associated with a 1·80 (1·34–2·27) percentage point change in predicted CVD risk, whereas each 10 μg/m^3^ change in PM_10_ concentration was associated with a 1·24 (0·84–1·63) percentage point change and each 10 μg/m^3^ change in O_3_ concentration with a 0·58 (0·33–0·83) percentage point change. Overall, the observed changes in air pollutants during the study period were associated with an average 6·6 percentage point reduction in predicted CVD risk.

**Interpretation:**

The CCAA and improved air quality were associated with a slower increase in predicted CVD risk, supporting the necessity for stricter, multipollutant air quality policies to maximise public health benefits.

**Funding:**

National Natural Science Foundation of China, Kadoorie Charitable Foundation, Noncommunicable Chronic Diseases-National Science and Technology Major Project, National Key R&D Program of China, Chinese Ministry of Science and Technology, and UK Wellcome Trust.

## Introduction

Cardiovascular disease (CVD) has been the leading cause of death globally for over 20 years, with more than 80% of cardiovascular deaths in 2021 occurring in low-income and middle-income countries.^1^^,^^2^ In addition to well established lifestyle risk factors, the large burden of CVD in low-income and middle-income countries could be attributed largely to high concentrations of ambient air pollutants.^3^ Long-term exposure to particulate matters (PMs) and ozone (O_3_) has been linked to increased CVD risk^4^, ^5^, ^6^, ^7^, ^8^ and exacerbation of CVD risk factors.^9^, ^10^, ^11^, ^12^ In China, more than 99·9% of the population was exposed to PM_2·5_ concentrations and 98·5% was exposed to O_3_ concentrations exceeding the 2021 WHO Air Quality Guideline limits.^13^ In 2019, 1·2 million CVD deaths were attributed to ambient PM exposure and 1·5 million CVD deaths to O_3_ exposure,^2^^,^^14^ emphasising the importance of implementing clean air policies and evaluating their potential benefits for reducing CVD burden.

**Research in context**

**Evidence before this study**

We searched PubMed, Embase, and Google Scholar from database inception to June 1, 2026, without language restrictions, for studies evaluating the association between clean air policies or improvements in air quality and cardiovascular disease (CVD). Key search terms were “clean air policy” OR “air pollution control” OR “air quality improvement” OR “changes in air pollution”, AND “cardiovascular disease”. Evidence from high-income countries suggests that clean air policies potentially reduce cardiovascular mortality and improve air quality. China’s Clean Air Act (CCAA), implemented in 2013, has been associated with substantial reductions in particulate matter concentration over the past decade. Previous studies have linked this reduction to a decreased CVD burden. Given the potentially heterogeneous effects of this policy on different pollutants and its broader public health impacts, evaluating its overall association with CVD risk provides more comprehensive insights than focusing on individual pollutants alone. Furthermore, because previous studies have relied on ecological designs, a causal inference framework using individual-level data is warranted to improve methodological rigour and validity.

**Added value of this study**

Using repeated individual-level measurements from a large nationwide cohort and a quasi-experimental difference-in-differences design, we found that the implementation of CCAA was associated with a marked decrease in PM_2·5_ and PM_10_ concentrations, accompanied by a modest increase in O_3_ levels. CCAA implementation was associated with a slower rate of CVD risk progression, with greater benefits observed under stricter enforcement. Furthermore, reductions in particulate matter concentrations were strongly associated with lower CVD risk, whereas increases in O_3_ levels were linked to increased CVD risk. Overall, the observed changes in air pollutants during the study period were associated with a net reduction in predicted CVD risk.

**Implications of all the available evidence**

Taken together, our findings suggest that the implementation of clean air policies might contribute to reducing cardiovascular risk at the population level. Our findings highlight the need for continuous, integrated pollution control strategies to maximise public health gains, providing a valuable reference for low-income and middle-income countries.

Previous evaluations of changes in disease risk following the implementation of clean air policies have primarily relied on ecological studies. Studies conducted in high-income countries have shown that reductions in air pollutant concentrations resulting from clean air policies (such as the 1990 Clean Air Act in the USA) were associated with reductions in CVD mortality and emergency hospital admissions.^15^, ^16^, ^17^, ^18^ In 2013, the State Council of China implemented China’s Clean Air Act (CCAA), one of the most stringent air pollution control policies at the time. Between 2013 and 2017, a series of measures to optimise energy structures and reduce emissions resulted in a 32% reduction in national average PM_2·5_ concentrations.^19^ A previous study assessing the health impacts of CCAA implementation estimated that this reduction was associated with 28 220 fewer premature deaths from ischaemic heart disease (IHD) and stroke in China.^20^ Although this analysis provided crucial evidence regarding the reduced disease burden associated with pollution control, it did not directly assess the association between changes in pollution levels and CVD risk. Similar ecological studies were limited by inferences made based on aggregate-level data and were more susceptible to confounding bias.^21^^,^^22^ Furthermore, because PM_2·5_ can scavenge O_3_ precursors, such as hydroperoxyl radicals, reductions in PM_2·5_ concentrations following CCAA implementation might have contributed to increases in O_3_ concentrations,^23^ which has been associated with an increase in the number of CVD incident cases.^24^ Given the complex effects of CCAA implementation on multiple pollutants and its potential broader effects on economic activity, employment, and other societal factors,^25^ it remains unclear whether this policy is associated with a reduction in individual-level CVD risk.

A quasi-experimental difference-in-difference (DID) approach, leveraging improvements in air quality and repeated measurements from a population-based cohort, provides a more rigorous framework for evaluating the association between air pollution control policies and CVD-related outcomes. A recent study using a DID analysis reported an association between CCAA implementation and improved lipid profiles.^26^ However, directly applying the DID framework to incident CVD outcomes presents methodological challenges when estimating the effects of policy interventions on disease risk. Established CVD risk prediction models provide a practical alternative by estimating future CVD risk based on multiple risk factors.^27^, ^28^, ^29^, ^30^ Predicted 10-year CVD risk is widely used for risk stratification and clinical decision making in China, the USA, and Europe.^27^, ^28^, ^29^ Therefore, these validated models offer an opportunity to assess the potential influence of air pollution control policies on future CVD risk through policy-induced changes in intermediate risk factors.^31^

Capitalising on the quasi-experimental setting created by the CCAA, we used baseline and resurvey data from the China Kadoorie Biobank (CKB) to evaluate the association of CCAA implementation with predicted CVD risk and its underlying risk factors.

## Methods

### Study population

The CKB study is a prospective cohort study, and its design has been described in detail elsewhere.^32^ Between 2004 and 2008, the CKB recruited 512 724 participants from ten provinces across China, including five urban (Qingdao, Harbin, Haikou, Suzhou, and Liuzhou) and five rural (Chengdu, Tianshui, Xinxiang, Jiaxing, and Changsha) areas. After the baseline survey, approximately 5% of the remaining participants were selected for repeated surveys by cluster sampling to participate in repeated resurveys at approximately 5-year intervals (appendix p 3). To date, three resurveys have been conducted (in 2008, 2013–14, and 2020–21). At each survey, trained staff administered electronic questionnaires with built-in quality control procedures and conducted physical examinations and blood sampling. The study was approved by the Ethical Review Committee of the Chinese Center for Disease Control and Prevention (Beijing, China), Peking University Health Science Center (Beijing, China), and Oxford Tropical Research Ethics Committee, University of Oxford (UK). All participants provided written informed consent before data collection.

### Study design

This prospective, quasi-experimental study included two parts of analyses (appendix p 24). First, we applied a quasi-experimental DID analysis to evaluate the associations between CCAA implementation and CVD risk. The standard two-way fixed-effects DID model relies on ordinary least squares estimation. When applied to binary or time-to-event outcomes, such as disease incidence, this approach might violate the assumption of linear parallel trends, thereby compromising the validity of causal inference.^33^ Furthermore, incorporating high-dimensional fixed effects into nonlinear models might hinder coefficient estimation or introduce bias.^34^, ^35^, ^36^ Therefore, we used predicted 10-year CVD risk as an alternative outcome.

We excluded participants who did not participate in any resurvey, had CVD at baseline, or had missing data on study variables at baseline or across all resurveys (n=477 862), leaving 34 862 participants who participated in at least one resurvey and contributed 100 763 observations to the analysis (study population 1). The number of participants in each survey wave is shown in the appendix (p 25). To mimic a controlled trial, we classified the ten study provinces into intervention and control groups based on the PM reduction targets set during CCAA implementation. Specifically, the Chinese Government established a national goal of reducing PM concentrations by 10% under the CCAA framework. Provincial governments subsequently set their own targets aligned with this benchmark (appendix p 10). Following established methodologies from previous studies,^26^^,^^37^, ^38^, ^39^ provinces that set targets for at least a 10% reduction in either PM_10_ or PM_2·5_ concentrations were designated as intervention areas, and participants from these provinces were assigned to the intervention group.

Second, we performed an explanatory analysis, examining the associations between changes in PM_2·5_, PM_10_, and O_3_ concentrations after CCAA implementation and changes in predicted CVD risk. A total of 15 521 individuals who participated in both the 2013–14 and 2020–21 resurveys were included (study population 2).

### Assessment of PM and O_3_ concentrations

Details of the PM and O_3_ concentration assessments have been published elsewhere.^40^, ^41^, ^42^, ^43^ Random-forest models were used to predict daily concentrations of PM_2·5_, PM_10_, and maximum daily 8-h average O_3_ concentrations at a spatial resolution of 1 km across mainland China from 2005 to 2021. The models were developed using ground measurements of these pollutants as dependent variables. Independent variables included both general predictors (such as meteorological parameters, land-use data, and population density) and pollutant-specific predictors. For PM_2·5_ and PM_10_, Multi-Angle Implementation of Atmospheric Correction aerosol optical depth and Modern-Era Retrospective Analysis for Research and Applications Version 2 PM_2·5_ and PM_10_ estimates were included. For O_3_, Community Multiscale Air Quality O_3_ simulations were included.^40^ The ten-fold cross-validated R^2^ values were 0·85 for PM_2·5_, 0·87 for PM_10_, and 0·80 for O_3_. The root-mean-square errors were 15·29 μg/m^3^ for PM_2·5_, 22·07 μg/m^3^ for PM_10_, and 22·93 μg/m^3^ for O_3_. Annual average concentrations of pollutants were calculated by averaging the daily concentrations.

The predicted pollutant concentrations for each 1 × 1 km grid cell were then assigned to participants according to the latitude and longitude of the clinics where they participated in the baseline survey. Given that these clinics were located within the participants’ administrative units and that most participants resided within approximately 1 km of their local clinics, this approach provides reliable estimates of community-level exposure.^44^, ^45^, ^46^, ^47^, ^48^

### Assessment of CVD predictors and covariates

The questionnaire collected data on participants’ sociodemographic characteristics, lifestyle factors, disease and medication history, family history, and exposure to second-hand smoke. Data on indoor pollution were obtained by asking about the fuel type used for cooking and heating. Height, weight, waist circumference, and blood pressure were measured with calibrated instruments and following standardised procedures. BMI was calculated by dividing weight (kg) by the square of standing height (m). We considered ecological covariates to account for potential clustering effects by area, including annual per capita gross domestic product, population size, and number of hospital beds per 1000 people at the province level, sourced from statistical yearbooks. The city’s average daily temperature on the survey date was obtained from historical reanalysis datasets provided by the National Aeronautics and Space Administration. Details on the assessment and categorisations of predictors and covariates are provided in the appendix (pp 3–4).

### Assessment of outcomes

The primary outcomes of this study were the predicted 10-year risks of CVD, IHD, ischaemic stroke, and haemorrhagic stroke, calculated using the CKB-CVD model, a non-laboratory-based risk prediction model developed for the CKB population.^30^ The predicted 10-year risk represents the probability of developing the corresponding disease within the following 10 years and theoretically ranges from 0 to 100%. Predictors included age, systolic blood pressure (SBP), diastolic blood pressure (DBP), use of antihypertensive medications, current smoking status, self-reported diabetes, and waist circumference. The model has good predictive performance, with Harrell’s C index ranging from 0·73 to 0·80.^30^ The predicted risks were calculated using predictor levels, disease-specific coefficients, and baseline survival estimates provided by the model (appendix pp 4–5, 11). Individual predicted 10-year CVD risks were calculated at four timepoints, including baseline and the three subsequent resurveys, incorporating changes in risk factors to update the risk estimates over time. Secondary outcomes were intermediate CVD risk factors, including SBP, DBP, BMI, and waist circumference.

### Statistical analysis

In the first analysis, we used the DID model to evaluate the association between CCAA implementation and predicted CVD risk. This method leverages the differences in predicted risks between the intervention and control groups and the differences between the period before and after policy implementation to isolate the policy effect from potential confounding (appendix p 5).^37^^,^^49^, ^50^, ^51^, ^52^ We applied the following model:

(1) $$\log\left(Y_{ijt}\right)=\beta \times {AIE}_{it}+\sum \beta_{z}\times Z+\lambda_{i}+\pi_{t}+\varepsilon_{ijt}$$

where $Y_{ijt}$ indicates the predicted risks of participant *i* in province *j* on survey date *t*. The predicted risks were log-transformed to follow a normal distribution. ${AIE}_{it}$ is the exposure of interest, which was derived from two dimensions: a geographical dimension, defined by the classification of intervention and control groups and a temporal dimension, determined by whether the CCAA had been implemented at the time of each survey. ${AIE}_{it}$ was estimated by including an interaction term in the model:

(2) $${AIE}_{it}={\mathrm{Int}}_{i}\;\times \;{After}_{t}$$

where ${\mathrm{Int}}_{i}$ indicates whether the individual *i* was assigned to the intervention group (1=yes, 0=no), and ${After}_{t}$ indicates whether the survey occurred after the CCAA implementation date (Sept 10, 2013; 1=yes, 0=no). β represents the average intervention effect of CCAA implementation. $\left(e^{\beta }-1\right)\times 100\%$ is interpreted as the percentage change in predicted risks in the intervention groups compared with the control groups (net change). Z and $\beta_{z}$ represent four sets of covariates and their corresponding coefficients, with model 4 as the final and primary model (appendix pp 5–6). Covariates were selected based on previous literature on ambient air pollution, health policy, and CVD.^37^^,^^52^^,^^53^ $\lambda_{j}$ is the fixed effect for individuals, accounting for time-invariant confounders specific to each participant (eg, sex and region) and within-individual correlation. $\pi_{t}$ is the fixed effect for study years, controlling for confounders that are common to all participants in a given year (eg, the COVID-19 pandemic), and the temporal trends in predicted risk. $\varepsilon_{ijt}$is the error term. To test the parallel trends assumption, we evaluated whether changes in predicted risks differed between the two groups before CCAA implementation (appendix p 6).^52^ Subgroup analyses were performed by age, sex, household income, and education level. The heterogeneity of estimates between subgroups was examined using the χ^2^ test. The DID model in equation 1 was also used to estimate the associations between CCAA implementation and SBP, DBP, BMI, and waist circumference without log-transforming these risk factors.

In the second analysis, we estimated the associations between each 10 μg/m^3^ change in pollutant concentrations and the change in predicted outcome risk between the 2013–14 and 2020–21 resurveys. In this analysis, the variation of interests was the changes in pollutant concentrations, which were calculated as.

$$\Delta Pollutant={Pollutant}_{2013-14}-{Pollutant}_{2020-21,}$$

and the changes in predicted risks were calculated as

$$\Delta Risk={Risk}_{2013-14}-{Risk}_{2020-21.}$$

As the changes in predicted risks approximated normal distributions, linear models with covariates that were consistent with those in model 4 were fitted (appendix pp 5–6). We also estimated the associations separately for rural and urban areas, with the interaction between pollution changes and region type assessed using the likelihood ratio test. The relationships between changes in pollutants and predicted risks were plotted using restricted cubic splines.

Based on the associations between changes in pollutants and predicted risks and the amount of pollutant changes, we estimated how the predicted risks would be distributed in the 2020–21 resurvey without pollutant changes after CCAA implementation (appendix pp 6–7). We used a similar approach to assess the associations between changes in PM concentrations and CVD risk factors.

Sensitivity analyses for the DID model and the explanatory analysis are detailed in the appendix (pp 7–8). All statistical analyses were performed using R (version 4.3.3) and Stata (version 17.0).

### Role of the funding source

The funders had no role in the study design, data collection, data analysis and interpretation, writing of the report, or the decision to submit the article for publication.

## Results

### Study population

Among 34 862 individuals in the study population 1, 13 252 (38·0%) participated in one resurvey, 12 181 (34·9%) participated in two resurveys, and 9429 (27·0%) participated in all three resurveys. The mean (SD) age of study population 1 at baseline was 51·3 (10·1) years, and 21 524 (61·7%) participants were female (table). The intervention group included 25 497 participants who lived in areas with a target of reducing PM concentration by 10% or more, whereas the control group included 9365 participants. The control group showed higher levels of education, household income, and consumption of fresh fruits and red meat and included a higher proportion of individuals with cookstove ventilation. In contrast, the intervention group had a higher prevalence of current smoking, heavy alcohol consumption, solid fuel use, and hypertension and a higher level of total physical activity. Participants in study population 1 had similar baseline characteristics to those in study population 2 and the full CKB cohort (appendix p 12).

**Table:** Baseline characteristics of study population 1

	Total population (N=34 862)	Intervention group (n=25 497)	Control group (n=9365)
Age, years	51·3 (10·1)	51·1 (10·1)	51·9 (10·1)
Female	21 524 (61·7%)	15 573 (61·1%)	5951 (63·5%)
High school and above	7128 (20·4%)	2724 (10·7%)	4404 (47·0%)
Household income ≥10 000 RMB/year	24 019 (68·9%)	16 193 (63·5%)	7826 (83·6%)
Current smoker	7945 (22·8%)	6294 (24·7%)	1651 (17·6%)
Heavy alcohol consumer∗	3450 (9·9%)	2831 (11·1%)	619 (6·6%)
Daily consumer of			
Fresh fruits	6787 (19·5%)	3419 (13·4%)	3368 (36·0%)
Fresh vegetables	33 397 (95·8%)	24 284 (95·2%)	9113 (97·3%)
Red meat	11 253 (32·3%)	5227 (20·5%)	6026 (64·3%)
Total physical activity, MET-h/day	21·3 (14·1)	23·1 (14·8)	16·4 (10·5)
Leisure sedentary time, h/week	21·3 (10·7)	21·0 (10·7)	22·2 (10·7)
Cooking with solid fuels	12 131 (34·8%)	11 628 (45·6%)	503 (5·4%)
Heating with solid fuels	13 115 (37·6%)	12 616 (49·5%)	499 (5·3%)
Having cookstove ventilation	15 476 (44·5%)	8350 (32·8%)	7126 (76·2%)
Duration of living with a smoker, years	22·4 (18·1)	23·6 (18·3%)	19·1 (16·8%)
Duration of exposure to second-hand smoke, h/week	6·9 (10·1)	7·3 (10·4%)	5·9 (9·1%)
BMI, kg/m^2^	23·7 (3·4)	23·6 (3·4)	24·0 (3·2%)
Waist circumference, cm	79·7 (9·6)	79·3 (9·5)	80·8 (9·5%)
Systolic blood pressure, mmHg	130·4 (20·9)	132·1 (20·9)	125·8 (20·0%)
Diastolic blood pressure, mmHg	77·4 (11·0)	78·0 (11·0)	75·7 (11·0%)
Prevalence of hypertension	11 687 (33·5%)	9020 (35·4%)	2667 (28·5%)
Taking antihypertensive medications	3682 (10·6%)	2722 (10·7%)	960 (10·3%)
Self-reported history of diabetes	910 (2·6%)	545 (2·1%)	365 (3·9%)
Self-rated good health	16 556 (47·5%)	12 556 (49·2%)	4000 (42·7%)
Family history of CVD	7292 (20·9%)	5079 (19·9%)	2213 (23·6%)

Data are n (%) or mean (SD). CVD=cardiovascular disease. MET=metabolic equivalent. RMB=Renminbi.

∗ Heavy alcohol consumers refer to individuals with alcohol consumption of ≥30 g/day or former weekly drinkers.

Between 2005 and 2013, both the intervention and control groups had persistently high concentrations of PM_2·5_, PM_10_, and O_3_ (figure 1). However, following the implementation of CCAA in 2013, PM_2·5_ concentrations decreased by 50·3% in the intervention group and 47·6% in the control group between 2013 and 2021. Over the same period, PM_10_ concentrations decreased by 49·0% in the intervention group and by 46·1% in the control group, whereas O_3_ concentrations increased by 22·2% in the intervention group and by 29·3% in the control group. Although the intervention group had continuously higher concentrations of pollutants than the control group from 2005 to 2021, the gap narrowed for PMs and expanded for O_3_ after 2013.

**Figure 1:**
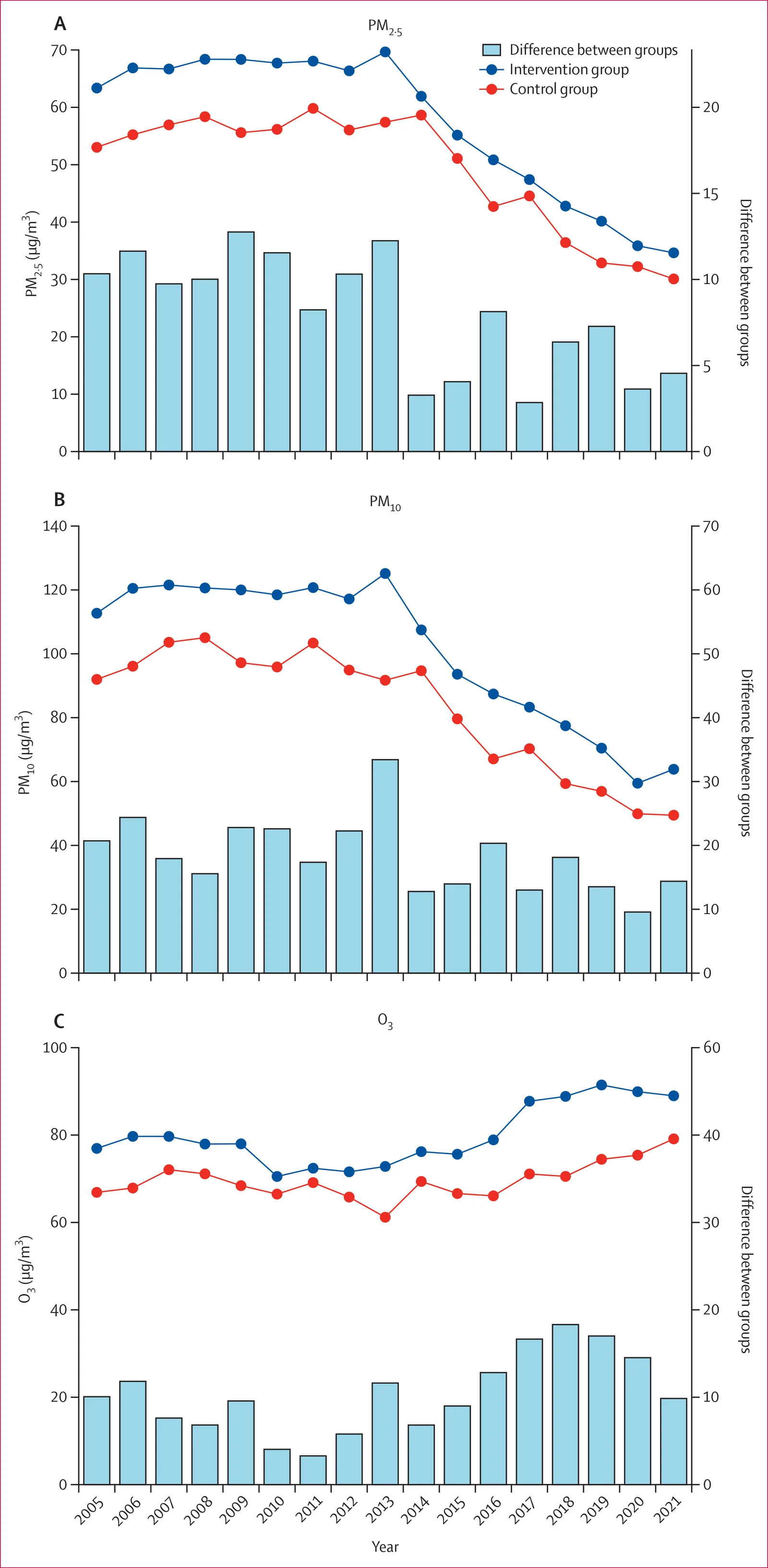
Temporal change in pollutant concentrations in the intervention and control groups from 2005 to 2021 PM=particulate matter.

As participants aged, most CVD risk factors, except for current smoking, progressively increased (appendix pp 13–14, 26). Consequently, the predicted CVD risks of the study participants increased with the increasing number of resurveys (appendix p 27). The increasing trends of predicted CVD risk in both groups were parallel between the baseline and 2008 resurveys, but the intervention group experienced smaller increases in the last two resurveys.

### Implementation of CCAA and predicted CVD risk

After CCAA implementation, the intervention group experienced lower increases in the predicted risks of CVD and its subtypes than the control group, with the predicted risks being lower by 3·95% (95% CI 3·18–4·72%) for CVD, 2·02% (1·38–2·65%) for IHD, 5·77% (4·88–6·64%) for ischaemic stroke, and 8·57% (7·18–9·93%) for haemorrhagic stroke after adjusting for all covariates (figure 2; all p<0·0001, model 4). When the cutoff was set to 12% or more, the results remained almost unchanged. However, defining the intervention group with a target of 15% or more resulted in greater differences between the two groups. The sensitivity analysis conducted without the observations from the 2013–14 resurvey also yielded larger effect estimates. The IHD results were not statistically significant when including individuals who participated in all the resurveys. The application of the Poisson Pseudo Maximum Likelihood estimator, mixed effects model, or inverse probability weighting did not substantially alter the results. For CVD, IHD, and ischaemic stroke, the increasing trends in predicted risk did not differ between the two groups before policy implementation (appendix p 15). Although a pre-policy difference was observed for haemorrhagic stroke, the honest DID analysis showed that the CI for haemorrhagic stroke did not include zero, even at $\bar{M}$=2 (appendix p 16). This observation indicated that our main results remained robust to allowing violations of the parallel trends assumption at least twice as large as the observed pre-policy differences.

**Figure 2:**
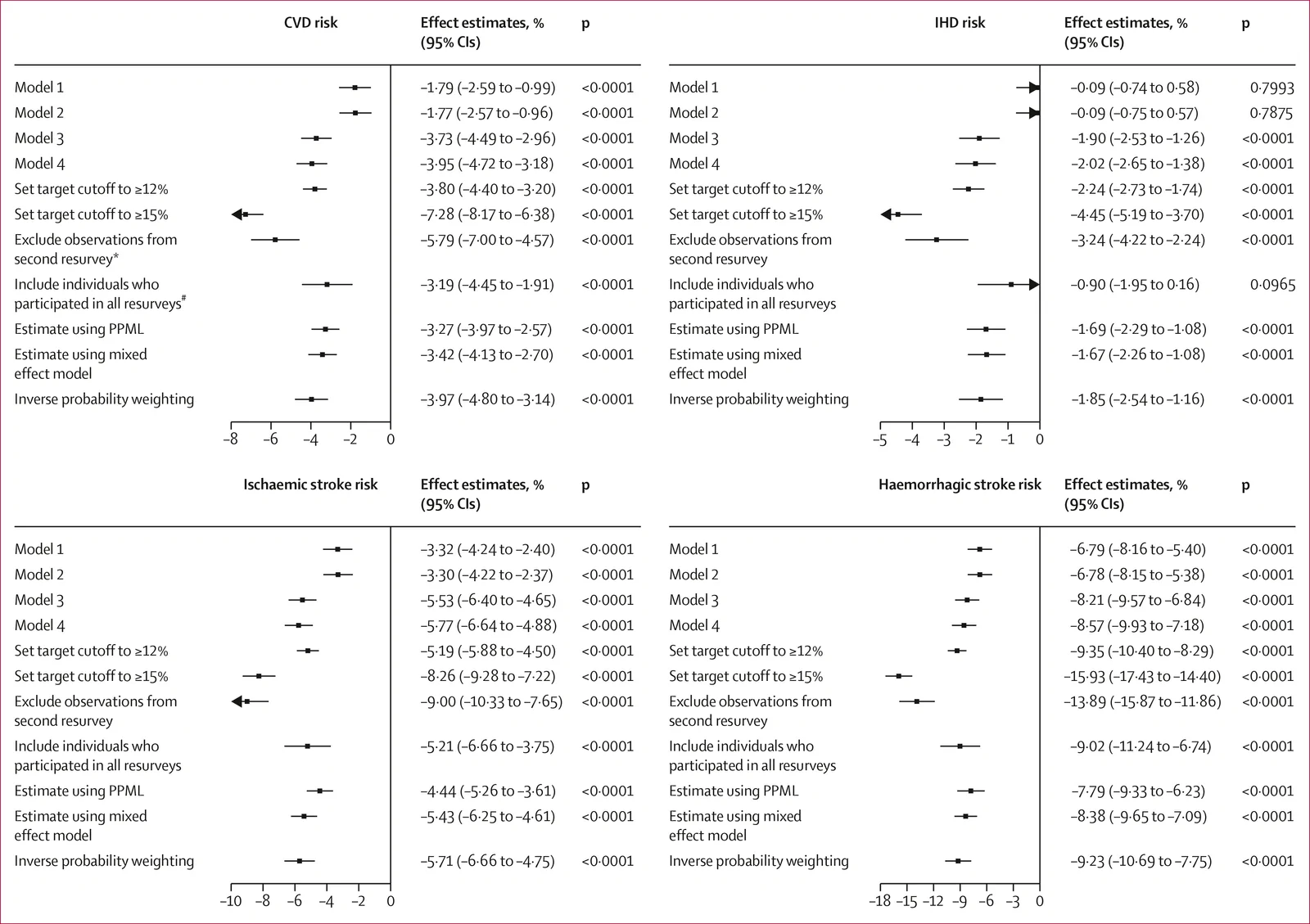
Estimated associations of China’s Clean Air Act implementation with predicted cardiovascular disease risk Model 1 was adjusted for age, gross domestic product per capita, population size, hospital beds per 1000 people, and average temperatures on the survey date. Model 2 was additionally adjusted for education level and household income. Model 3 was additionally adjusted for alcohol consumption, intakes of red meat, fresh fruits, and fresh vegetables, total physical activity level, leisure sedentary time, BMI, self-rated health status, and family histories of heart disease and stroke. Model 4 was additionally adjusted for fuel types for cooking and heating, stove ventilation, duration of living with a smoker, and exposure to second-hand smoke. ∗After excluding observations from the 2013–14 resurvey, 31 176 individuals with 72 974 observations remained. #A total of 9429 individuals with 37 716 observations participated in all three resurveys. CVD=cardiovascular disease. IHD=ischaemic heart disease. PPML=Poisson Pseudo Maximum Likelihood.

Most associations between CCAA implementation and predicted risks did not differ significantly across age, sex, income, or education level (figure 3). However, the associations with predicted CVD and ischaemic stroke risk were greater in older participants (age ≥60 years). The association with predicted IHD risk was stronger among individuals with higher education levels, whereas the association with predicted haemorrhagic stroke risk was more evident in female participants. In the intervention group, we observed net decreases in SBP, DBP, and waist circumference but a slight increase in BMI (appendix p 17).

**Figure 3:**
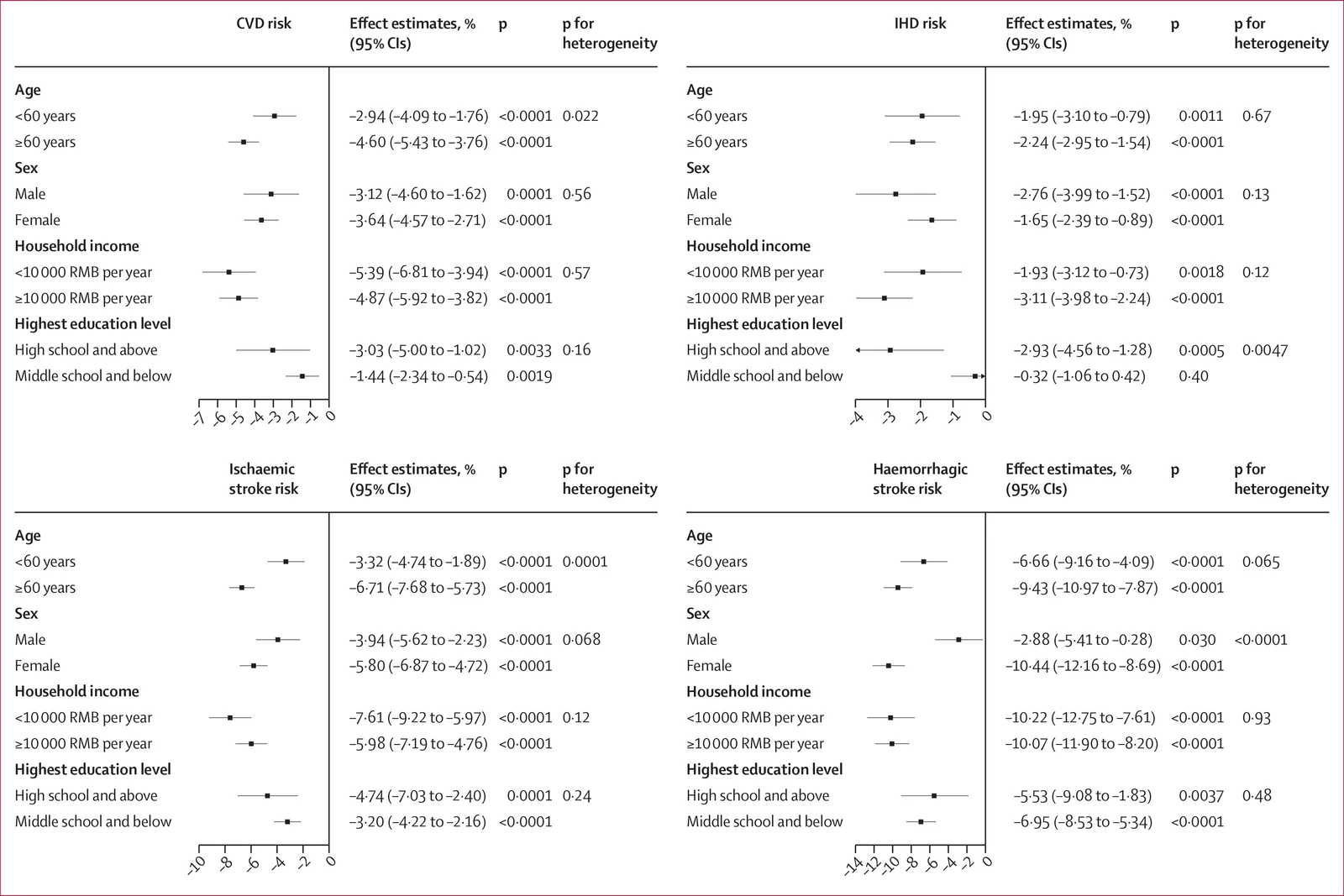
Subgroup analyses of the estimated associations of China’s Clean Air Act implementation with predicted cardiovascular disease risk The covariates adjusted in the model are the same as those in Model 4 of figure 2, except for the subgroup characteristics. CVD=cardiovascular disease. IHD=ischaemic heart disease. RMB=Renminbi.

### Changes in pollutants and predicted CVD risk

Between the 2013–14 and 2020–21 resurveys, changes in pollutant concentrations were positively linked to changes in predicted outcome risks (figure 4). A 10 μg/m^3^ change in PM_2·5_ concentrations was associated with a 1·80 (1·34–2·27) percentage point change in predicted CVD risk. The corresponding changes were 1·24 (0·84–1·63) percentage points for PM_10_ concentrations and 0·58 (0·33–0·83) percentage points for O_3_ concentrations. Similar but weaker associations were observed for CVD subtypes, except that the association between O_3_ concentration changes and predicted IHD risk was not statistically significant. The associations between changes in PM concentrations and predicted risks were linear (appendix pp 28–29), but those between changes in O_3_ concentrations and CVD, ischaemic stroke, and haemorrhagic stroke were J-shaped (appendix p 30).

**Figure 4:**
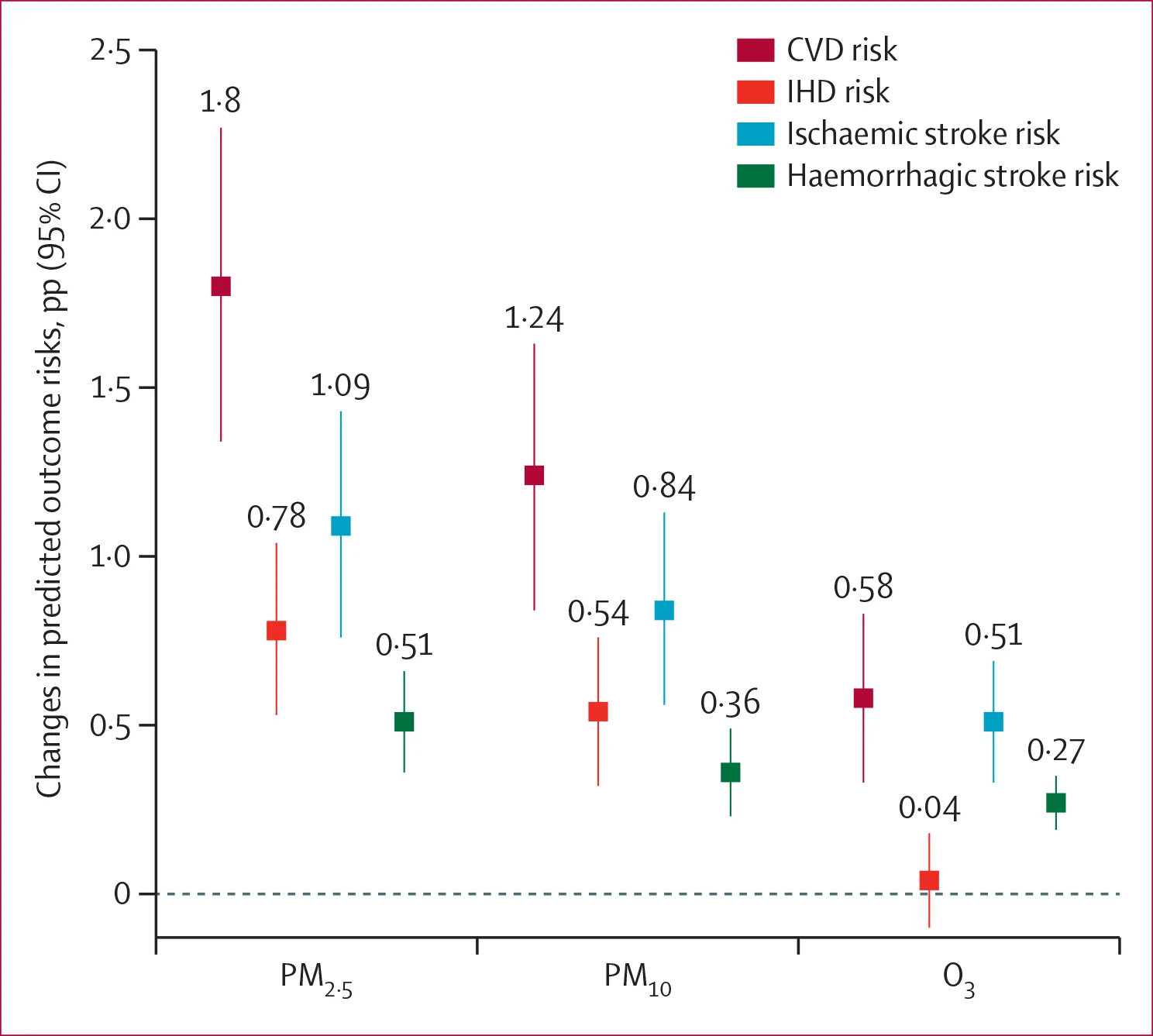
Associations between changes in pollutant concentrations (per 10 μg/m^3^) and predicted cardiovascular disease risks The changes in air pollutant concentrations and the predicted outcome risks were calculated as the differences between the concentrations at the 2013–14 and 2020–21 resurveys. The covariates adjusted in the models are the same as those in Model 4 of figure 2 but with an additional adjustment for sex and the corresponding pollutant concentration at the 2013–14 resurvey. Except for the temperatures, which are from the 2020–21 and 2013–14 resurveys, all covariates are from the 2013–14 resurvey. CVD=cardiovascular disease. IHD=ischaemic heart disease. pp=percentage point. PM=particulate matter.

To quantify the associations in terms of SD changes, we performed Z-score normalisation to the predicted risk and observed similar positive associations of changes in pollutant concentrations with predicted risks of CVD, ischaemic stroke, and haemorrhagic stroke but statistically insignificant associations of changes in PM_10_ and O_3_ concentrations with predicted IHD risk (appendix p 31). A 10% relative change in PM_2·5_ concentrations was associated with a 0·83 (0·52–1·15) percentage point change in predicted CVD risk, whereas the corresponding changes were 0·59 (0·15–1·02) percentage points for PM_10_ concentrations and 0·51 (0·34–0·67) percentage points for O_3_ concentrations. Similar associations were observed for most predicted risks of CVD subtypes (appendix p 32). In the two-pollutant model, the effect estimates for PM concentrations and the predicted risks slightly decreased but remained statistically significant after adjusting for O_3_ concentrations (appendix p 19). However, after adjusting for PM concentrations, most associations with O_3_ concentrations were not statistically significant. In the sensitivity analysis using a time-varying Cox model, decreases in PM_2·5_ and PM_10_ concentrations were generally associated with reduced CVD risk, although some of the associations were not significant, likely due to the limited number of cases (appendix p 20). The effect estimates for PM_2·5_ and PM_10_ concentrations were larger in rural areas, whereas those for O_3_ concentrations were larger in urban areas (appendix p 21). Given the associations with predicted CVD risks, in the absence of the decreases in PM_2·5_ and PM_10_ concentrations and the increase in O_3_ concentrations, the predicted CVD risk in the 2020–21 resurvey would have increased by an average of 6·6 percentage points. The corresponding changes were 3·3 percentage points for IHD, 4·0 percentage points for ischaemic stroke, and 2·4 percentage points for haemorrhagic stroke (appendix pp 33–34).

Changes in PM and O_3_ concentrations were positively associated with changes in SBP, DBP, and BMI in most single-pollutant or two-pollutant models (appendix pp 22–23).

## Discussion

In this prospective, quasi-experimental study, CCAA implementation was followed by a gradual decrease in the annual average PM exposure and a concurrent increase in O_3_ concentrations. We observed that CCAA implementation was associated with a slower rate of increase in predicted 10-year risks of CVD, IHD, ischaemic stroke, and haemorrhagic stroke over time. In the explanatory analyses, we further found that during 2013–21, reductions in PM concentrations were associated with reductions in predicted CVD risks, whereas increases in O_3_ concentrations were associated with increases in predicted CVD risks. However, the combined changes in air pollutants during this period were associated with a net reduction in predicted risks. Similar beneficial associations of CCAA implementation and its accompanying reductions in air pollutant concentrations were also observed for several CVD risk factors.

Previous studies on the health benefits of the 1990 Clean Air Act in the USA have shown that the clean air policy was followed by reduction in CVD mortality and emergency hospital admissions over subsequent decades.^15^, ^16^, ^17^ An ecological study based on air quality monitoring and mortality data from 74 key cities in China estimated that decreases in PM_2·5_ concentrations between 2013 and 2017 were associated with the prevention of 8220 premature deaths from IHD and 20 000 premature deaths from stroke.^20^ A national case-crossover study in 292 Chinese cities similarly estimated that reductions in PM_2·5_ concentrations between 2013 and 2017 were associated with an approximately 30% lower attributable fraction of cardiac arrhythmias, coronary heart disease, heart failure, and stroke.^22^ Another study also reported that reductions in PM concentrations in Beijing were associated with a lower relative risk of CVD mortality.^21^ However, these studies are subject to the inherent limitations of ecological studies and did not investigate the aetiological pathways linking air pollution control to cardiovascular outcomes. Our study addressed these limitations by incorporating a DID analysis based on individual-level data. In the DID analysis, we used multiple resurvey data and a counterfactual framework to better control individual-level confounders and their secular changes,^50^ thereby increasing confidence in the causal link between pollutant regulation and improvements in CVD risk factors and predicted CVD risk. Notably, the estimated associations increased when higher intervention cutoffs were used. Our findings suggest that greater governmental commitment and more stringent implementation of pollution control policies can substantially improve population-level cardiovascular health outcomes.

Substantial evidence has demonstrated that CVD risk factors mediate the association between air pollution and CVD risk.^9^^,^^54^, ^55^, ^56^, ^57^, ^58^ In this study, we used predicted CVD risk, a composite measure derived from established risk factors, to assess the association between air pollution control and CVD risk. Compared with observed CVD events, predicted CVD risk more effectively captures the potential effects of air pollution control on future CVD risk through changes in risk factors. This approach provides a novel framework for evaluating similar environmental policies or broader macro-level interventions. In regions where disease surveillance systems are lacking or underdeveloped, repeated measurement of individual-level risk factors can serve as a feasible method for estimating disease risk and assessing the public health implications of policy implementation.

Further explanatory analyses showed that reductions in PM concentrations were associated with lower predicted CVD risk, whereas increases in O_3_ concentrations were associated with higher predicted risk, consistent with previous cohort studies reporting positive associations between both PMs and O_3_ concentrations and CVD risk.^4^, ^5^, ^6^, ^7^, ^8^ A recent quasi-experimental analysis of 247 496 participants from the ChinaHEART study showed that changes in PM_2·5_ and O_3_ concentrations attributable to the CCAA were positively associated with changes in blood lipid concentrations, thereby supporting our findings.^26^ Similarly, a Canadian study on the impact of a 10-year shift in ambient air quality on mortality indicated that the long-term stability of O_3_ concentrations restricted the health benefits of overall air quality improvement between 2007 and 2016.^59^ Nevertheless, after considering both the association effects and magnitudes of pollutant changes between 2013 and 2021, our findings suggest that the net effect attenuated the expected upward trajectory of predicted 10-year CVD risk that would otherwise be expected with ageing. Under the assumptions of our analytical model, the observed improvements in air quality during this period were associated with an average 6.6 percentage point smaller increase in predicted 10-year CVD risk. The observed improvements correspond to approximately 66 fewer predicted CVD events per 1000 individuals over the decade following 2021.

We found that the associations of CCAA implementation might have stronger effect estimates among older adults, women, and individuals with higher educational levels. Previous studies have also noted stronger associations between PM exposure and CVD in older adults,^5^^,^^6^ possibly reflecting a deterioration in adaptive immune function in response to environmental stressors.^60^ Our findings underscore the importance of the clean air policies for protecting vulnerable populations. However, previous evidence regarding differences by sex and educational attainment in the association between PM exposure and CVD has been inconsistent,^6^^,^^44^^,^^61^ indicating a need for further research.

Both CCAA implementation and changes in air pollutants were associated with lower blood pressure. A systematic review and meta-analysis of 41 cross-sectional and longitudinal studies provided evidence linking long-term PM exposure to elevated blood pressure.^54^ Longitudinal and cohort studies have also linked O_3_ exposure to elevated blood pressure and hypertension.^62^^,^^63^ The mediation by blood pressure might explain the association between CCAA implementation and predicted CVD risk. Furthermore, the varied effects of blood pressure on various CVD outcomes could explain the stronger association between CCAA implementation and stroke than between CCAA implementation and IHD. A meta-analysis of randomised controlled trials discovered that blood pressure reduction has a greater effect on reducing the risk of stroke than on reducing the risk of IHD.^64^ Mendelian randomisation studies have also revealed that blood pressure has a stronger association with haemorrhagic stroke than with major coronary events and ischaemic stroke.^65^ We observed paradoxical associations of CCAA with BMI and waist circumference. The differential findings likely reflect the different physiological implications of these measures, with BMI reflecting lean and fat mass, whereas waist circumference more directly reflects central adiposity. Therefore, CCAA implementation might be linked to decreased central obesity and increased lean body mass. Nevertheless, given the small magnitude of the policy’s effect sizes on both metrics, this result should be interpreted cautiously.

Several studies have evaluated the health benefits of short-term air quality improvements resulting from emission controls during large-scale sporting events or international conferences.^66^, ^67^, ^68^, ^69^ These studies consistently found that short-term reductions in pollutant concentrations were associated with improvements in cardiac autonomic function,^66^ inflammation and oxidative stress,^67^^,^^68^ and thrombosis–endothelial dysfunction,^69^ supporting the biological plausibility of our findings.

To our knowledge, this is the first study to use individual-level repeated measurements to evaluate the association between a national clean air policy and predicted CVD risk. This study has several strengths. First, the CKB study collected extensive information on potential confounders, rigorous adjustment for known confounders in the model (eg, indoor air pollution). Furthermore, incorporating a counterfactual analysis feature helped to partially address unmeasured confounding and their secular changes. Second, high-resolution air pollutant data enabled us to link changes in air pollution to predicted risks, providing potential explanations for the intervention effects observed in the group comparisons. Finally, the broad geographical distribution of the study population and the wide range of pollutant exposures enhance the generalisability of the findings.

Some limitations should be acknowledged. First, some degree of prediction error in estimating CVD risk was inevitable. However, given the robust performance of the risk prediction model, these errors are likely random and would bias the effect estimates towards the null. Sensitivity analysis based on observed CVD cases also indicated a protective association of PM reduction. Second, allocating the study population to the intervention and control groups based on regional air pollution control targets could introduce potential regional confounding and clustering effects into our analysis. Nevertheless, our DID model effectively accounts for time-invariant confounders and common temporal trends shared by both groups. Furthermore, we carefully adjusted for time-varying regional and individual-level confounders in the analysis. Sensitivity analysis using a mixed effect model yielded highly consistent estimates, indicating that our primary analysis is robust against within-region correlation. Third, other policies implemented during the study period might have served as temporal confounders. However, the CCAA was the principal air pollution control initiative in China during this period, encompassing a comprehensive set of regulatory and enforcement measures. Consequently, most air quality management efforts implemented in this period were performed under the framework of the CCAA. To our knowledge, no national or provincial measures during this time, apart from the CCAA, had been widely recognised and possibly associated with both regional differences in implementation targets and reductions in CVD and its risk factors. Fourth, our findings could be sensitive to the cutoff value used for group classification. However, the sensitivity analysis using progressively higher cutoffs yielded larger effect estimates. Fifth, attrition of participants during resurveys differed between the intervention and control groups. Nevertheless, sensitivity analyses restricted to the complete cases yielded consistent results, indicating that our results were not substantially affected by attrition bias. Lastly, pollutant exposures were assigned based on the clinics nearest to the participants’ residences, which could have led to non-differential misclassification and underestimated associations.

In conclusion, implementation of the CCAA in 2013 and subsequent reductions in PM concentrations were associated with slower increases in CVD risk. However, increases in O_3_ concentrations over the same period might have diminished the policy’s protective association. These findings add to the evidence linking PM and O_3_ concentrations with CVD risk and suggest that CCAA implementation might contribute to improved cardiovascular health. Our study provides empirical evidence supporting the potential cardiovascular health benefits of pollution control and strengthens the rationale for adopting and optimising clean air policies in China and other low-income and middle-income countries. Our findings support the necessity of enforcing more stringent clean air policies and promoting coordinated control of multiple pollutants. In 2023, China’s State Council issued the Continuous Improvement of Air Quality Action Plan, which established more ambitious targets for reducing air pollution. Future research should evaluate the implications of this new policy.

## Data sharing

Details on how to access China Kadoorie Biobank data and details of the data release schedule are available from the China Kadoorie Biobank website.

## Declaration of interests

We declare no competing interests.
