# Supplementary material for: Exploring China’s Clean Air Act and associated cardiovascular disease risk: a prospective, quasi-experimental, and causal inference modelling study
Source: Lancet Planet Health. 2026 Sep 1;10(8):None. doi: 10.1016/j.lanplh.2026.101513 (PMC13627081; doi:10.1016/j.lanplh.2026.101513)
Supplement: Supplementary file 1 [file mmc1.pdf]

THE LANCET
Planetary Health

Supplementary appendix

This appendix formed part of the original submission and has been peer reviewed.
We post it as supplied by the authors.

Supplement to: Sun D, Meng X, Hu J, et al. Exploring China's Clean Air Act and associated cardiovascular disease risk: a prospective, quasi-experimental, and causal inference modelling study. *Lancet Planet Health* 2026. https://doi.org/10.1016/j.lanplh.2026.101513

Appendix

Members of the China Kadoorie Biobank collaborative group: ... 2
Supplementary methods ... 3
Sampling procedure for the CKB resurvey ... 3
Assessment and categorization of predictors and covariates ... 3
Calculation of the predicted risks ... 4
Difference-in-difference model ... 5
Model adjustments ... 5
Tests for parallel trends assumption ... 6
Predicted risk in the absence of changes in pollutant ... 6
Sensitivity analysis for the DID model ... 7
Sensitivity analysis for explanatory analysis ... 7
Supplementary results ... 10
Table S1. Particulate matter reduction targets and study area assignments ... 10
Table S2. Predictors and their coefficients of the CKB-CVD model ... 11
Table S3. Baseline characteristics of the study populations and the full CKB cohort ... 12
Table S4. Characteristics of the participants in each resurvey ... 13
Table S5. Tests of parallel trend assumptions before the CCAA implementation ... 15
Table S6. Honest DID analysis ... 16
Table S7. Estimated effects of the implementation of the CCAA on the intermediate risk factors for CVD ... 17
Table S8. Effect estimates (95% CIs) of changes in pollutants (per 10 μg/m^3) on the predicted CVD risks ... 18
Table S9. Associations between changes in pollutants (per 10 μg/m^3) and the predicted CVD risks in the two-pollutant model ... 19
Table S10. Association of annual changes in pollutants (per 10 μg/m^3) with the CVD risks using the time-varying Cox model ... 20
Table S11. Effect estimates (95% CIs) of changes in pollutants (per 10 μg/m^3) on the predicted CVD risks in rural and urban areas ... 21
Table S12. Associations between changes in pollutants (per 10 μg/m^3) and the intermediate risk factors for CVD ... 22
Table S13. Associations between changes in pollutants (per 10 μg/m^3) and the predicted CVD risks and intermediate risk factors for CVD in the two-pollutant model ... 23
Figure S1. Study design and participants selection ... 24
Figure S2. Participants in different survey wave ... 25
Figure S3. Distribution of intermediate risk factors in each survey ... 26
Figure S4. Temporal change of predicted CVD risks in the intervention and control groups ... 27
Figure S5. Concentration-response relationship between decreases in $PM_{2.5}$ and the predicted CVD risks ... 28
Figure S6. Concentration-response relationship between decreases in PM10 and the predicted CVD risks ... 29
Figure S7. Concentration-response relationship between increases in O_3 and the predicted CVD risks ... 30
Figure S8. Associations between changes in pollutants (per 10 μg/m^3) and per standard deviation change of the predicted cardiovascular disease risks ... 31
Figure S9. Associations between relative changes in pollutants (per 10% changes) and the predicted cardiovascular disease risks ... 32
Figure S10. Predicted outcome risks in the real scenario (2020-2021 resurvey) and counterfactual scenario in the absence of changes in $PM_{2.5}$, PM_{10}, and O_3 ... 34

Members of the China Kadoorie Biobank collaborative group:

International Steering Committee: Junshi Chen, Zhengming Chen (PI), Robert Clarke, Rory Collins, Liming Li (PI), Jun Lv, Richard Peto, Robin Walters.

International Co-ordinating Centre, Oxford: Daniel Avery, Maxim Barnard, Derrick Bennett, Ruth Boxall, Ka Hung Chan, Yiping Chen, Zhengming Chen, Charlotte Clarke, Johnathan Clarke; Robert Clarke, Huaidong Du, Ahmed Edris Mohamed, Hannah Fry, Simon Gilbert, Pek Kei Im, Andri Iona, Maria Kakkoura, Christiana Kartsonaki, Kshitij Kolhe, Hubert Lam, Kuang Lin, James Liu, Mohsen Mazidi, Iona Millwood, Sam Morris, Qunhua Nie, Alfred Pozarickij, Maryam Rahmati, Paul Ryder, Dan Schmidt, Becky Stevens, Iain Turnbull, Robin Walters, Baihan Wang, Lin Wang, Neil Wright, Ling Yang, Xiaoming Yang, Pang Yao.

National Co-ordinating Centre, Beijing: Jun Lv, Canqing Yu, Dianjianyi Sun, Yuanjie Pang, Can Hou, Qingmei Xia, Chao Liu, Pei Pei, Lang Pan, Xiao Han, Honglu Bian, Xinxin Chen.

10 Regional Co-ordinating Centres: Qingdao CDC: Zengchang Pang, Ruqin Gao, Shanpeng Li, Haiping Duan, Shaojie Wang, Yongmei Liu, Ranran Du, Liang Cheng, Xiaocao Tian, Hua Zhang. **Licang CDC:** Dan Hu, Xiaoyan Zheng, Yujie Wang. **Heilongjiang Provincial CDC:** Wei Sun, Shichun Yan, Xiaoming Cui. **Nangang CDC:** Chi Wang, Zhenyuan Wu, Lishun Zhai, Zhaoxi Pang, Shiwen Dong. **Hainan Provincial CDC:** Huiming Luo, Jinyan Chen, Bin He, Dingwei Sun, Xingren Wang, Tingting Ou. **Meilan CDC:** Xiangyang Zheng, Dewei Zheng, Shuai Yang, Yilei Li, Lihui Li, Xingjiao Chen. **Jiangsu Provincial CDC:** Jinyi Zhou, Ran Tao, Jian Su, Xikang Fan, Zongming Cheng, Yuxiao Huang. **Suzhou CDC:** Yan Lu, Yujie Hua, Li Xing, Shuxian Wang, Jianrong Jin, Juping Ma, Jinchao Liu, Kaifei Zhu, Hongfu Ren, Xingfeng Shen. **Guangxi Provincial CDC:** Ge Zhong, Wei Mao, Zhenzhen Lu, Ling He. **Liuzhou CDC:** Lifang Zhou, Changping Xie, Jian Lan, Tingping Zhu, Jinxue Tan, Liuping Wei, Liyuan Zhou, Sisi Wang. **Sichuan Provincial CDC:** Xianping Wu, Ningmei Zhang, Xiaofang Chen, Xiaoyu Chang, Zhuo Wang, Yujin He. **Pengzhou CDC:** Mingqiang Yuan, Xia Wu, Xiaofang Chen, Zhaodong Wang, Qiang Sun, Yang Lin. **Gansu Provincial CDC:** Faqing Chen, Xiaolan Ren, Lijun Chang, Feiming Zhong. **Maiji CDC:** Jianjun Feng, Weijie Hu, Xiaofang Zhang, Yalin Chen, Fei Wang, Jun Wang. **Henan Provincial CDC:** Linqi Diao, Wanshen Guo, Zhiwei Han, Dongyang Zhao, Dengjun Zhu, Kai Kang, Shixian Feng, Huizi Tian, Yali Yan, Bing Han, Li Gao, Shaofang Li, Huafei Feng, Wei Tang. **Huixian CDC:** Xiaolin Li, Huarong Sun, Xiaocong Zhao, Ying Li, Chen Hu, Pan He, Xukui Zhang, Yuanyuan Jin, Hesheng Zhang. **Zhejiang Provincial CDC:** Min Yu, Ruying Hu, Hao Wang, Weiwei Gong, Jieming Zhong, Meng Wang, Chunxiao Xu, Keqing Gong. **Tongxiang CDC**: Hao Xu, Yuan Cao, Kaixu Xie, Lingli Chen, Xiaomei Tu,Chen Chen. **Hunan Provincial CDC:** Xiaojun Li, Li Yin, Huilin Liu, Yuan Liu, Yi Liu, Lei Yin, Xian Xie, Jing Wang. **Liuyang CDC:** Bo Xiao, Pingsheng Lou, Yuan Peng, Libo Zhang, Chan Qu, Qili Jiang, Yanling Chen, Yan Zhao.

Supplementary methods

Sampling procedure for the CKB resurvey

In the first resurvey (2008 resurvey), we used the administrative unit as the primary sampling unit and conducted cluster random sampling across 10 CKB study areas, enrolling approximately 25,000 participants (about 5% of the cohort). For the subsequent two resurveys, the same recruitment target of 25,000 participants was maintained. Priority was given to individuals who had participated in previous resurveys; if the target was not met, additional participants were recruited through continued cluster random sampling from the remaining CKB population who have complete baseline data[1]. In these resurveys, participants were recruited from the same administrative unit as those included in the baseline survey.

Assessment and categorization of predictors and covariates

Predictors in the CKB-CVD model include age (years), SBP (mmHg), DBP (mmHg), taking antihypertensive medications (yes or no), current daily smoking (yes or no), self-reported diabetes (yes or no), and WC (cm). Following a minimum of 5 minutes of rest, trained staff took two BP readings on each participant's right upper arm. If the SBP readings differed by more than 10 mmHg, a third measurement was performed. The average of the last two readings was used for analysis. Participants who were taking antihypertensive medications were those who reported a diagnosed hypertension still on treatment or reported taking drugs to lower blood pressure. Current daily smokers were participants who reported smoking every day. Self-reported diabetes was defined as a diabetes diagnosed by a physician before each survey. WC was measured at the midpoint between the iliac crest and the lower rib margin at the end of normal expiration using flexible plastic tape.

Educational levels were divided into no formal, primary, middle, and high school or higher. Annual household income was categorized as <10,000 RMB, 10,000-19,999 RMB, and ≥20,000 RMB. For alcohol consumption, we inquired about the usual frequency of drinking, the preferred type of alcoholic beverage, and the amount of alcohol typically consumed on a drinking day in the past 12 months. We then categorized alcohol consumption into six categories: never or less than weekly drinkers, former weekly drinkers, weekly but not daily drinkers, and daily drinkers, with daily

intake levels further classified as less than 30 g, 30-59 g, and 60 g or more of pure alcohol per day. We used a validated qualitative food frequency questionnaire to assess the regular consumption of 12 conventional food groups in the past 12 months[2]. The weekly frequencies of consuming red meat, fresh fruits, and fresh vegetables were determined by the midpoint value of each frequency category: daily (7), 4-6 days/week (5), 1-3 days/week (2), monthly (0.5), and never/rarely (0). We asked about the usual type and duration of activities related to work, commuting, domestic, and leisure-time in the past 12 months. Total physical activity level, expressed in metabolic equivalent task-hours per day (MET-hours/day), was computed by multiplying the duration (in hours/day) of each activity by its MET value, and then summing up the MET-h/d for all activities. Leisure sedentary time was defined as the time (in hours/week) spent watching TV or reading. Self-rated health was classified into excellent, good, fair, and poor. Family histories of heart disease and stroke were defined as having at least one parent or sibling with the corresponding disease. Participants were asked to report their hours of second-hand smoke exposure per week and the years they had lived with a smoker. Participants were surveyed on their cooking frequency and home heating habits during winter. Those with in-home cooking facilities were asked about the presence of stove ventilation. Additionally, we asked participants who cooked monthly or heated their homes in the winter about their primary cooking and heating fuels: gas, coal, wood, electricity, and others. Coal and wood were categorized as solid fuels, whereas gas, electricity, and central heating were categorized as clean. Fuel types for cooking and heating were classified into three groups: clean fuels, solid fuels, and other fuels or no monthly cooking or heating during winter.

Calculation of the predicted risks

The development and validation of the CKB-CVD model have been published[3]. Briefly, we developed 10-year risk prediction models for IHD, IS, and HS, separately, using data from 289,464 females and 200,132 males without baseline CVD in the CKB cohort. The model development involved two steps: predictor selection and coefficient estimation. First, we constructed a base model that included baseline age, SBP, current daily smoking, and self-reported diabetes, along with interaction terms between age and each of the other three predictors. Subsequently, other well-established CVD risk factors were added to the model one by one. Using the relative integrated discrimination index (rIDI) as the criterion for incremental performance, we identified

antihypertensive medication use, DBP, and waist circumference as additional predictors. The coefficients of the predictors and the 10-year baseline survival rate were estimated using Cox proportional hazards models. Model performance, as assessed by the Harrell's C index, was evaluated by internal-external cross-validation across the ten study areas[3].

The 10-year risk of the three CVD subtypes were estimated by following formula:

$$Risk_i = 1 - S_i^{\exp(\sum \beta_{ij} \times x_j)}$$

where i indicates the three outcomes (i.e. IHD, IS, or HS), S_i is the disease-specific baseline survival estimate at 10 years, and $\sum \beta_{ij} \times x_j$ is the linear predictor, calculated by summing the product of the predictors' levels and their disease-specific coefficients. The baseline survival and the coefficients were provided in Table S2. The 10-year CVD risk was calculated as:

$$Risk_{CVD} = 1 - (1 - Risk_{IHD}) \times (1 - Risk_{IS}) \times (1 - Risk_{HS})$$

Validation has shown that $Risk_{CVD}$ calculated from this formula agreed with the estimates after considering the competing risk of these three subtypes or using CVD as a single outcome[3,4].

Difference-in-difference model

In contrast to the conventional observational study, which leverages the variation in exposure level, the DID analysis estimates the effect of exposure by using changes over time in an intervention group relative to a control group. Specifically, DID enables within-individual comparison before and after the intervention (the first difference), thereby controlling for time-invariant confounders. These within-individual changes are further compared between the intervention and control groups (the second difference) to account for common temporal trends, including the effect of time and time-varying confounders.

Model adjustments

The DID models were adjusted stepwise. The model 1 was adjusted for age, GDP per capita, population, hospital beds per 1000 people, and average temperatures on the survey date. The model 2 was further adjusted for education level and household income. The model 3 was additionally adjusted for alcohol consumption, intakes of red

meat, fresh fruits, and fresh vegetables, total physical activity level, leisure sedentary time, BMI, self-rated health status, and family histories of heart disease and stroke. The model 4 was further adjusted for fuel types for cooking and heating, stove ventilation, duration of living with a smoker, and exposure to second-hand smoke.

In the second explanatory analysis, we applied linear models with adjustments for sex, levels of the corresponding pollutant at the 2013-14 resurvey, and the same covariates in Model 4 were fitted. Except for temperature, which was from the 2013-14 and 2020-21 resurveys, all other covariates were from the 2013-14 resurvey. The three pollutants were not mutually adjusted in the main analysis.

Tests for parallel trends assumption

One key assumption of DID analysis is that the intervention and control groups would have parallel trends in predicted risk in the absence of the CCAA. Although we cannot observe the counterfactual, we tested this assumption by comparing the trends in predicted risk for the two groups before the CCAA. We fitted the following model:

$$log(Y_{ijt}) = \sum_{m=k,m\neq 2}^{M} \beta_k \times D\_Int_{jt,k} + \sum \beta_z \times Z + \lambda_i + \pi_t + \varepsilon_{ijt} \qquad (2)$$

where $D\_Int_{jt,k}$ are a set of dummy variables representing the intervention status at different surveys, M is the times of survey, which is 4, and $m \neq 2$ indicates that the estimated effect of CCAA at each survey (β_k) were relative to the 2008 resurvey. Since both the baseline survey and the 2008 resurvey were conducted before the CCAA, β_k should be close to 0 when k=1.

Additionally, we conducted a sensitivity analysis using the honest DID approach[5]. Rather than assuming strict parallel trends, this method evaluates whether the results remain robust under violations of this assumption. Specifically, it tests whether the results remain statistically significant even when allowing the post-intervention trend deviation to be up to $\bar{M}$ times the magnitude of the pre-intervention deviation. We evaluated the policy effect at the 2020-21 resurvey under varying values of $\bar{M}$ (0, 0.5, 1, 1.5, and 2), where $\bar{M}$ =1 serves as the natural benchmark, as recommended by the original methodology paper.

Predicted risk in the absence of changes in pollutant

In the counterfactual scenario where pollutant levels remained unchanged, the risk reduction attributable to changes in pollutant levels should be added back. If the

estimated associations were causal, the predicted CVD risks in 2020-21 resurvey would be calculated as:

$$Risk_{counterfactual} = Risk_{real} + \sum \beta_{\Delta Pollutant_i} \times \Delta Pollutant_i$$

where $Risk_{real}$ is the observed predicted risks in the 2020-21 resurvey, $\Delta Pollutant_i$ indicates the changes in the three pollutants ($PM_{2.5}$, PM_{10}, and O_3), calculated as $\Delta Pollutant = Pollutant_{2013-14} - Pollutant_{2020-21}$, and $\beta_{\Delta Pollutant_i}$ indicates the estimated associations between these pollutant changes and the corresponding risk reductions between the 2013-14 and 2020-21 resurveys.

Sensitivity analysis for the DID model

To assess the sensitivity of the results to grouping, we changed the target cutoff to ⩾12% and ⩾15%. In addition, we removed observations from the 2013-14 resurvey because it was within the policy transition period. Because the participants in different resurveys were partially different, we performed a sensitivity analysis by restricting the analysis in the individuals who participated in all resurveys to prevent any differential attrition between the two groups. We also applied the Poisson Pseudo Maximum Likelihood (PPML) estimation to relax the requirement for statistical independence in the error term[6]. Mixed effect model with nested random intercepts for individuals and study areas were used to further account for within-individual correlation and clustering effect. To further account for regional confounding and the endogeneity issue it caused, we performed the inverse probability weighting (IPW) approach based on the propensity score[7]. Propensity scores were estimated using individual-level exposure to average concentrations of $PM_{2.5}$, PM_{10}, and O_3, as well as average levels of GDP per capita, population size, hospital beds per 1,000 people, and temperature aggregated over the period from 2004 to 2013.

Sensitivity analysis for explanatory analysis

In the sensitivity analysis, we assessed the associations of per 10% change in pollutants with the changes in predicted risks. We also performed two-pollutant models to mutually adjust for PMs and O_3. $PM_{2.5}$ and PM_{10} were not adjusted for each other due to their high correlation (Pearson r^2=0.97).

Moreover, we leveraged the incident CVD cases during follow-up to assess the

impact of changes in pollutants. This analysis included participants without prevalent CVD from the 2013-14 resurvey (n=24,105). CVD morbidity and mortality were ascertained through linkage with the national health insurance database, inpatient medical record database, and local disease and death registry systems. Outcomes were defined as incident IHD (ICD-10: I20-I25), IS (I63), HS (I62), and composite CVD (defined as the occurrence of any incident IHD, IS, and HS) during follow-up. Follow-up time was censored at the earliest occurrence of CVD onset, death, loss to follow-up, or 31 December 2022, whichever came first. We split the follow-up data into annual intervals to evaluate the changes in pollutants in a time-varying manner. We applied a time-varying Cox proportional hazard model to assess the association between annual changes in pollutant concentrations and CVD risk. The main exposure was the annual changes in pollutant concentration, calculated as the average level in the previous year minus the level in the current year, consistent with the definition used in previous studies[8,9]. The pollutant levels in the previous year were adjusted for in the model, together with the other covariates in the models of Figure 4 of the manuscript.

Reference

1. Chen, Z., Chen, J., Collins, R., Guo, Y., Peto, R., Wu, F., Li, L., and China Kadoorie Biobank collaborative, g. (2011). China Kadoorie Biobank of 0.5 million people: survey methods, baseline characteristics and long-term follow-up. Int. J. Epidemiol. *40*, 1652-1666. 10.1093/ije/dyr120.
2. Zhu, N., Yu, C., Guo, Y., Bian, Z., Han, Y., Yang, L., Chen, Y., Du, H., Li, H., Liu, F., et al. (2019). Adherence to a healthy lifestyle and all-cause and cause-specific mortality in Chinese adults: a 10-year prospective study of 0.5 million people. Int J Behav Nutr Phys Act *16*, 98. 10.1186/s12966-019-0860-z.
3. Yang, S., Han, Y., Yu, C., Guo, Y., Pang, Y., Sun, D., Pei, P., Yang, L., Chen, Y., Du, H., et al. (2022). Development of a Model to Predict 10-Year Risk of Ischemic and Hemorrhagic Stroke and Ischemic Heart Disease Using the China Kadoorie Biobank. Neurology *98*, e2307-e2317. 10.1212/wnl.0000000000200139.
4. Group, W.C.R.C.W. (2019). World Health Organization cardiovascular disease risk charts: revised models to estimate risk in 21 global regions. Lancet Glob Health *7*, e1332-e1345. 10.1016/S2214-109X(19)30318-3.
5. Rambachan, A., and Roth, J. (2023). A more credible approach to parallel trends. Rev. Econ. Stud. *90*, 2555-2591.
6. Ciani, E., and Fisher, P. (2019). Dif-in-Dif Estimators of Multiplicative Treatment Effects. Journal of Econometric Methods *8*. 10.1515/jem-2016-0011.
7. Austin, P.C., and Stuart, E.A. (2015). Moving towards best practice when using inverse probability of treatment weighting (IPTW) using the propensity score to estimate causal treatment effects in observational studies. Stat. Med. *34*, 3661-3679. 10.1002/sim.6607.
8. Bo, Y., Brook, J.R., Lin, C., Chang, L.Y., Guo, C., Zeng, Y., Yu, Z., Tam, T., Lau, A.K.H., and Lao, X.Q. (2021). Reduced Ambient PM(2.5) Was Associated with a Decreased Risk of Chronic Kidney Disease: A Longitudinal Cohort Study. Environ. Sci. Technol. *55*, 6876-6883. 10.1021/acs.est.1c00552.
9. Downs, S.H., Schindler, C., Liu, L.J., Keidel, D., Bayer-Oglesby, L., Brutsche, M.H., Gerbase, M.W., Keller, R., Künzli, N., Leuenberger, P., et al. (2007). Reduced exposure to PM10 and attenuated age-related decline in lung function. New Engl. J. Med. *357*, 2338-2347. 10.1056/NEJMoa073625.

Supplementary results

Table S1. Particulate matter reduction targets and study area assignments

Provinces	Cities where the study areas are located	$PM_{2.5}$/PM_{10} reduction targets	Group assignments
Shandong	Qingdao	20% ($PM_{2.5}$)	Intervention group
Heilongjiang	Harbin	5% (PM_{10})	Control group
Hainan	Haikou	0	Control group
Jiangsu	Suzhou	20% ($PM_{2.5}$)	Intervention group
Guangxi	Liuzhou	5% (PM_{10})	Control group
Sichuan	Chengdu	10% (PM_{10})	Intervention group
Gansu	Tianshui	12% (PM_{10})	Intervention group
Henan	Xinxiang	15% (PM_{10})	Intervention group
Zhejiang	Jiaxing	20% ($PM_{2.5}$)	Intervention group
Hunan	Changsha	10% (PM_{10})	Intervention group

Table S2. Predictors and their coefficients of the CKB-CVD model

Sex	Predictors	IHD sub-model		IS sub-model		HS sub-model	
		Main effect	Interaction with age	Main effect	Interaction with age	Main effect	Interaction with age
Male	Age, years	0.0716554	/	0.0835158	/	0.0820196	/
	Systolic blood pressure, mmHg	0.0052816	0.0000168	0.0124603	-0.0004323	0.0168301	-0.0002353
	Diastolic blood pressure, mmHg	0.0091778	-0.0006826	0.0140617	-0.0003044	0.0287048	-0.0009919
	Taking antihypertensive medications	0.4660422	-0.0053771	0.4656016	-0.0116475	0.3949390	-0.0100336
	Current daily smoking	0.2024671	-0.0037854	0.2512960	-0.0050969	0.0962472	-0.0009236
	Self-reported diabetes	0.5971099	-0.0183296	0.6059128	-0.0127534	0.3715960	-0.0078866
	Waist circumference, cm	0.0125664	-0.0006205	0.0077610	-0.0001151	-0.0081349	-0.0004154
	Baseline survival estimated at 10 years	0.9287638	/	0.9275716	/	0.9825860	/
Female	Age, years	0.0666511	/	0.0740794	/	0.0820293	/
	Systolic blood pressure, mmHg	0.0022094	-0.0000259	0.0087970	-0.0004318	0.0147824	-0.0003590
	Diastolic blood pressure, mmHg	0.0052584	-0.0001553	0.0100736	-0.0001013	0.0280161	-0.0007176
	Taking antihypertensive medications	0.4381349	-0.0101449	0.4315641	-0.0089527	0.4797150	-0.0089115
	Current smoking	0.1601188	-0.0067592	0.2590549	-0.0155619	0.2458049	-0.0062883
	Self-reported diabetes	0.4633056	-0.0115995	0.5991041	-0.0208146	0.4047413	-0.0094574
	Waist circumference, cm	0.0146348	-0.0006227	0.0100013	-0.0004689	-0.0084531	-0.0001766
	Baseline survival estimated at 10 years	0.8996251	/	0.9103525	/	0.9858385	/

IHD, ischemic heart disease; IS, ischemic stroke; HS, hemorrhagic stroke.

Predictor variables were centered as follows: age at 55 years, systolic blood pressure at 120 mmHg, diastolic blood pressure at 80 mmHg, and waist circumference at 80 cm.

Table S3. Baseline characteristics of the study populations and the full CKB cohort

	Study population 1 (n=34,862) †	Study population 2 (n=15,521) ‡	Full cohort# (n=489,594)
Age, years (SD)	51.3 (10.1)	50.0 (9.1)	51.6 (10.6)
Female, n (%)	21,524 (61.7)	9,931 (64.0)	289,461 (59.1)
High school and above, n (%)	7,128 (20.4)	2,254 (14.5)	101,587 (20.7)
Household income≥10,000 RMB/year, n (%)	24,019 (68.9)	10,328 (66.5)	350,763 (71.6)
Current smokers, n (%)	7,945 (22.8)	3,432 (22.1)	120,091 (24.5)
Heavy alcohol consumers*, n (%)	3,450 (9.9)	1,501 (9.7)	52,306 (10.7)
Daily consumers of, n (%)			
Fresh fruits	6,787 (19.5)	2,062 (13.3)	89,560 (18.3)
Fresh vegetables	33,397 (95.8)	14,814 (95.4)	463,619 (94.7)
Red meat	11,253 (32.3)	3,754 (24.2)	142,214 (29.0)
Total physical activity, MET-hours/day (SD)	21.3 (14.1)	23.5 (14.4)	21.5 (13.9)
Leisure sedentary time, hours/week (SD)	21.3 (10.7)	21.1 (10.8)	21.0 (10.8)
Cooking with solid fuels, n (%)	12,131 (34.8)	6,707 (43.2)	170,205 (34.8)
Heating with solid fuels, n (%)	13,115 (37.6)	6,183 (39.8)	179,348 (36.6)
Having cookstove ventilation, n (%)	15,476 (44.5)	5,573 (36.0)	214,171 (43.8)
Duration of living with a smoker, years (SD)	22.4 (18.1)	23.4 (17.8)	22.7 (18.5)
Duration of exposure to second-hand smoke, hours/week (SD)	6.9 (10.1)	7.3 (10.3)	6.8 (9.7)
BMI, kg/m^2 (SD)	23.7 (3.4)	23.6 (3.3)	23.6 (3.4)
Waist circumference, cm (SD)	79.7 (9.6)	78.7 (9.1)	80.0 (9.7)
Systolic blood pressure, mmHg (SD)	130.4 (20.9)	130.4 (19.9)	130.6 (21.1)
Diastolic blood pressure, mmHg (SD)	77.4 (11.0)	77.8 (10.8)	77.7 (11.1)
Prevalence of hypertension, n (%)	11,687 (33.5)	5,069 (32.7)	165,163 (33.7)
Taking antihypertensive medications, n (%)	3,682 (10.6)	1,419 (9.1)	54,450 (11.1)
Self-reported history of diabetes, n (%)	910 (2.6)	249 (1.6)	13,312 (2.7)
Self-rated good health, n (%)	16,556 (47.5)	7,783 (50.1)	229,955 (47.0)
Family history of CVD, n (%)	7,292 (20.9)	3,192 (20.6)	97,378 (19.9)

SD, standard deviation; MET, metabolic equivalent of task; BMI, body mass index; CVD, cardiovascular disease.

Data are n (%) or mean (SD).

*Heavy alcohol consumers refer to individuals with a pure alcohol consumption of ≥30g/d or former weekly drinkers.

†Population 1 included the individuals who were without baseline CVD and participated in any resurvey.

‡Population 2 included the individuals who were without baseline CVD and participated in both the 2013-14 and 2020-21 resurveys.

# Participants with baseline CVD were excluded.

Table S4. Characteristics of the participants in each resurvey

	2008 resurvey			2013-14 resurvey			2020-21 resurvey		
	Total population (n=18,843)	Intervention group (n=14,433)	Control group (n=4,410)	Total population (n=24,105)	Intervention group (n=18,215)	Control group (n=5,890)	Total population (n=22,953)	Intervention group (n=18,550)	Control group (n=4,403)
Age, years (SD)	54.2 (10.5)	54.1 (10.4)	54.6 (10.9)	59.1 (10.1)	59.1 (10.0)	59.3 (10.3)	65.2 (9.0)	65.0 (9.1)	66.2 (8.7)
Female, n (%)	11,469 (60.9)	8,736 (60.5)	2,733 (62.0)	14,901 (61.8)	11,283 (61.9)	3,618 (61.4)	14,683 (64.0)	11,730 (63.2)	2,953 (67.1)
High school and above, n (%)	3,634 (19.3)	1,551 (10.7)	2,083 (47.2)	4,609 (19.1)	1,817 (10.0)	2,792 (47.4)	3,995 (17.4)	1,765 (9.5)	2,230 (50.6)
Household income≥10,000 RMB/year, n (%)	15,523 (82.4)	11,703 (81.1)	3,820 (86.6)	22,014 (91.3)	16,314 (89.6)	5,700 (96.8)	21,062 (91.8)	16,727 (90.2)	4,335 (98.5)
Current smokers, n (%)	4,212 (22.4)	3,453 (23.9)	759 (17.2)	4,566 (18.9)	3,653 (20.1)	913 (15.5)	3,687 (16.1)	3,212 (17.3)	475 (10.8)
Heavy alcohol consumers*, n (%)	1,806 (9.6)	1,515 (10.5)	291 (6.6)	2,644 (11.0)	2,091 (11.5)	553 (9.4)	2,606 (11.4)	2,158 (11.6)	448 (10.2)
Daily consumers of, n (%)									
Fresh fruits	3,699 (19.6)	1,798 (12.5)	1,901 (43.1)	8,148 (33.8)	4,853 (26.6)	3,295 (55.9)	9,227 (40.2)	6,188 (33.4)	3,039 (69.0)
Fresh vegetables	17,647 (93.7)	13,426 (93.0)	4,221 (95.7)	23,308 (96.7)	17,552 (96.4)	5,756 (97.7)	22,559 (98.3)	18,264 (98.5)	4,295 (97.5)
Red meat	4,416 (23.4)	1,832 (12.7)	2,584 (58.6)	10,340 (42.9)	6,404 (35.2)	3,936 (66.8)	7,880 (34.3)	5,741 (30.9)	2,139 (48.6)
Total physical activity, MET-hours/day (SD)	20.4 (13.8)	22.2 (14.3)	14.6 (10.1)	18.4 (13.8)	19.2 (14.3)	15.9 (11.6)	15.6 (13.7)	16.5 (14.4)	11.6 (9.0)
Leisure sedentary time, hours/week (SD)	20.4 (10.5)	20.4 (10.7)	20.3 (9.8)	30.4 (13.5)	30.6 (14.4)	30.4 (13.2)	27.0 (13.6)	29.1 (13.7)	26.5 (13.5)
Cooking with solid fuels, n (%)	6,170 (32.7)	6,029 (41.8)	141 (3.2)	4,354 (18.1)	4,200 (23.1)	154 (2.6)	3,078 (13.4)	3,057 (16.5)	21 (0.5)
Heating with solid fuels, n (%)	6,889 (36.6)	6,796 (47.1)	93 (2.1)	5,660 (23.5)	5,607 (30.8)	53 (0.9)	5,572 (24.3)	5,554 (29.9)	18 (0.4)
Having cookstove ventilation, n (%)	9,180 (48.7)	5,598 (38.8)	3,582 (81.2)	15,622 (64.8)	10,651 (58.5)	4,971 (84.4)	18,512 (80.7)	14,330 (77.3)	4,182 (95.0)
Duration of living with a smoker, years (SD)	25.5 (19.6)	27.5 (19.5)	19.1 (18.3)	15.4 (17.8)	17.0 (18.4)	10.5 (14.6)	19.5 (19.5)	20.2 (19.7)	16.8 (18.4)
Duration of exposure to second-hand smoke, hours/week (SD)	5.9 (8.6)	6.3 (8.8)	4.5 (7.8)	5.6 (10.5)	5.7 (10.4)	5.0 (10.8)	4.4 (8.8)	4.5 (8.9)	3.6 (8.3)
BMI, kg/m^2 (SD)	23.5 (3.4)	23.3 (3.4)	24.0 (3.3)	24.1 (3.5)	24.0 (3.5)	24.5 (3.5)	24.4 (3.5)	24.4 (3.5)	24.5 (3.4)

Waist circumference, cm (SD)	82.3 (10.0)	81.9 (10.1)	83.6 (9.5)	84.3 (9.9)	83.8 (9.9)	85.8 (10.0)	86.0 (9.7)	86.0 (9.7)	85.7 (9.6)
Systolic blood pressure, mmHg (SD)	133.2 (22.3)	134.7 (22.2)	128.3 (21.9)	139.5 (23.6)	140.5 (23.2)	136.3 (24.5)	139.0 (23.5)	139.2 (23.6)	138.2 (23.4)
Diastolic blood pressure, mmHg (SD)	78.4 (11.8)	79.3 (11.6)	75.6 (11.7)	80.6 (12.7)	81.2 (12.5)	78.7 (13.0)	82.1 (11.9)	82.3 (11.8)	81.1 (11.9)
Prevalence of hypertension, n (%)	5,421 (28.8)	4,301 (29.8)	1,120 (25.4)	11,763 (48.8)	8,710 (47.8)	3,053 (51.8)	13,043 (56.8)	10,599 (57.1)	2,444 (55.5)
Taking antihypertensive medications, n (%)	2,670 (14.2)	2,074 (14.4)	596 (13.5)	6,008 (24.9)	4,666 (25.6)	1,342 (22.8)	8,697 (37.9)	7,212 (38.9)	1,485 (33.7)
Self-reported history of diabetes, n (%)	773 (4.1)	504 (3.5)	269 (6.1)	1,690 (7.0)	1,171 (6.4)	519 (8.8)	2,832 (12.3)	2,180 (11.8)	652 (14.8)
Self-rated good health, n (%)	8,322 (44.2)	6,463 (44.8)	1,859 (42.2)	10,711 (44.4)	8,761 (48.1)	1,950 (33.1)	10,586 (46.1)	8,653 (46.6)	1,933 (43.9)
Family history of CVD, n (%)	3,888 (20.6)	2,815 (19.5)	1,073 (24.3)	5,474 (22.7)	3,925 (21.5)	1,549 (26.3)	6,721 (29.3)	5,139 (27.7)	1,582 (35.9)

SD, standard deviation; MET, metabolic equivalent of task; BMI, body mass index; CVD, cardiovascular disease.

Data are n (%) or mean (SD).

*Heavy alcohol consumers refer to individuals with a pure alcohol consumption of ≥30g/d or former weekly drinkers.

Table S5. Tests of parallel trend assumptions before the CCAA implementation

	Effect estimates, % (95% CIs)	*p*
CVD risk	-0.02 (-0.87, 0.84)	0.9711
IHD risk	0.66 (-0.03, 1.36)	0.0616
IS risk	-0.79 (-1.78, 0.20)	0.1157
HS risk	-1.63 (-3.18, -0.05)	0.0429

CVD, cardiovascular disease; IHD, ischemic heart disease; IS, ischemic stroke; HS, hemorrhagic stroke; CI, confidence interval.

The covariates adjusted in the model were the same as those in Model 4 of Figure 2.

Table S6. Honest DID analysis

Outcome	Effect estimates, %	$\overline{M}$	Lower CI	Upper CI
CVD	-5.89	0.5	-7.31	-4.45
		1	-7.80	-3.90
		1.5	-8.40	-3.25
		2	-9.02	-2.56
IHD	-3.14	0.5	-4.93	-1.70
		1	-6.02	-0.74
		1.5	-7.12	0.40
		2	-8.25	1.59
IS	-8.95	0.5	-10.73	-6.65
		1	-11.85	-5.25
		1.5	-13.13	-3.74
		2	-13.58	-2.18
HS	-15.36	0.5	-18.28	-11.60
		1	-20.21	-9.13
		1.5	-20.84	-6.45
		2	-20.84	-3.65

CVD, cardiovascular disease; IHD, ischemic heart disease; IS, ischemic stroke; HS, hemorrhagic stroke; CI, confidence interval.

Table S7. Estimated effects of the implementation of the CCAA on the intermediate risk factors for CVD

	Effect estimates (95% CIs)	*p*
Systolic blood pressure, mmHg	-5.05 (-5.59, -4.50)	<0.0001
Diastolic blood pressure, mmHg	-0.53 (-0.83, -0.22)	0.0007
BMI, kg/m^2 *	0.47 (0.42, 0.53)	<0.0001
Waist circumference, mm	-11.63 (-13.10, -10.15)	<0.0001

BMI, body mass index; CI, confidence interval.

The covariates adjusted in the model were the same as those in Model 4 of Figure 2.

* Body mass index was not adjusted for.

Table S8. Effect estimates (95% CIs) of changes in pollutants (per 10 μg/m^3) on the predicted CVD risks

Predicted risk, percentage point	$PM_{2.5}$		PM_{10}		O_3	
	Effect estimates (95% CIs)	*p*	Effect estimates (95% CIs)	*p*	Effect estimates (95% CIs)	*p*
CVD	1.80 (1.34, 2.27)	<0.0001	1.24 (0.84, 1.63)	<0.0001	0.58 (0.33, 0.83)	<0.0001
IHD	0.78 (0.53, 1.04)	<0.0001	0.54 (0.32, 0.76)	<0.0001	0.04 (-0.10, 0.18)	0.5483
IS	1.09 (0.76, 1.43)	<0.0001	0.84 (0.56, 1.13)	<0.0001	0.51 (0.33, 0.69)	<0.0001
HS	0.51 (0.36, 0.66)	<0.0001	0.36 (0.23, 0.49)	<0.0001	0.27 (0.19, 0.35)	<0.0001

CVD, cardiovascular disease; IHD, ischemic heart disease; IS, ischemic stroke; HS, hemorrhagic stroke; CI, confidence interval.

The changes in air pollutants and the predicted outcome risks were calculated as the differences between the levels at the 2013-14 and 2020-21 resurveys.

Table S9. Associations between changes in pollutants (per 10 μg/m^3) and the predicted CVD risks in the two-pollutant model

Predicted risk, percentage point	Adjusted pollutant*	$PM_{2.5}$		PM_{10}		O_3	
		Effect estimates (95% CIs)	*p*	Effect estimates (95% CIs)	*p*	Effect estimates (95% CIs)	*p*
CVD	O_3	1.62 (1.15, 2.09)	<0.0001	1.22 (0.77, 1.67)	<0.0001		
	$PM_{2.5}$					0.31 (0.02, 0.60)	0.0333
	PM_{10}					-0.26 (-0.64, 0.12)	0.1764
IHD	O_3	0.63 (0.37, 0.89)	<0.0001	0.52 (0.28, 0.77)	<0.0001		
	$PM_{2.5}$					0.01 (-0.15, 0.17)	0.9186
	PM_{10}					-0.19 (-0.40, 0.02)	0.0736
IS	O_3	0.95 (0.61, 1.30)	<0.0001	0.72 (0.40, 1.05)	<0.0001		
	$PM_{2.5}$					0.34 (0.13, 0.55)	0.0015
	PM_{10}					0.01 (-0.26, 0.28)	0.9314
HS	O_3	0.45 (0.30, 0.60)	<0.0001	0.32 (0.17, 0.46)	<0.0001		
	$PM_{2.5}$					0.13 (0.04, 0.22)	0.0057
	PM_{10}					-0.02 (-0.14, 0.10)	0.7459

CVD, cardiovascular disease; IHD, ischemic heart disease; IS, ischemic stroke; HS, hemorrhagic stroke; CI, confidence interval.

The changes in air pollutants and the predicted outcome risks were calculated as the differences between the levels at the 2013-14 and 2020-21 resurveys.

Except for the additional adjusted pollutants, the other covariates adjusted in the models were the same as those in the models of Figure 4.

*For each additionally adjusted pollutant, the change in pollutant levels between the two resurveys and the levels at the 2013-14 resurvey were included in the model. $PM_{2.5}$ and PM_{10} were not mutually adjusted due to high correlation (Pearson r^2=0.97).

Table S10. Association of annual changes in pollutants (per 10 μg/m^3) with the CVD risks using the time-varying Cox model

	Case	$PM_{2.5}$		PM_{10}		O_3	
		HR (95% CIs)	*p*	HR (95% CIs)	*p*	HR (95% CIs)	*p*
CVD	4,663	0.92 (0.85, 1.00)	0.0414	0.92 (0.88, 0.97)	<0.0001	1.00 (0.93, 1.09)	0.8994
IHD	2,725	0.95 (0.86, 1.05)	0.3253	0.95 (0.90, 1.01)	0.1079	1.08 (0.98, 1.20)	0.1115
IS	2,746	0.88 (0.79, 0.97)	0.0145	0.90 (0.84, 0.95)	0.0004	0.90 (0.82, 1.00)	0.0493
HS	496	0.92 (0.85, 1.00)	0.0414	0.96 (0.82, 1.12)	0.6316	0.99 (0.78, 1.25)	0.9118

CVD, cardiovascular disease; IHD, ischemic heart disease; IS, ischemic stroke; HS, hemorrhagic stroke; HR, hazard ratio; CI, confidence interval.

The annual changes in air pollutants were calculated according to the concentration in each year and the previous year.

The pollutant levels in the previous year were adjusted for in the model, together with the other covariates in the models of Figure 4.

Table S11. Effect estimates (95% CIs) of changes in pollutants (per 10 μg/m^3) on the predicted CVD risks in rural and urban areas

Predicted risk, percentage point	$PM_{2.5}$			PM_{10}			O_3		
	Rural	Urban	*p* for interaction	Rural	Urban	*p* for interaction	Rural	Urban	*p* for interaction
CVD	2.13 (1.60, 2.65)	0.91 (0.36, 1.46)	<0.0001	0.94 (0.43, 1.44)	0.55 (0.13, 0.96)	0.0281	0.06 (-0.38, 0.50)	1.49 (1.00, 1.97)	0.0003
IHD	0.88 (0.59, 1.17)	0.48 (0.17, 0.78)	0.0098	0.28 (-0.00, 0.56)	0.32 (0.09, 0.55)	0.6333	-0.48 (-0.73, -0.24)	0.74 (0.47, 1.01)	<0.0001
IS	1.28 (0.89, 1.66)	0.53 (0.13, 0.93)	0.0002	0.60 (0.23, 0.96)	0.39 (0.09, 0.69)	0.1075	0.06 (-0.26, 0.38)	1.23 (0.88, 1.58)	<0.0001
HS	0.58 (0.42, 0.75)	0.27 (0.09, 0.44)	0.0004	0.15 (-0.02, 0.31)	0.16 (0.02, 0.29)	0.8454	-0.02 (-0.16, 0.12)	0.70 (0.54, 0.85)	<0.0001

CVD, cardiovascular disease; IHD, ischemic heart disease; IS, ischemic stroke; HS, hemorrhagic stroke.

The changes in air pollutants and the predicted outcome risks were calculated as the differences between the levels at the 2013-14 and 2020-21 resurveys.

The covariates adjusted in the models were the same as those in models of Figure 4 in the manuscript.

Table S12. Associations between changes in pollutants (per 10 μg/m^3) and the intermediate risk factors for CVD

	$PM_{2.5}$		PM_{10}		O_3	
	Effect estimates (95% CIs)	*p*	Effect estimates (95% CIs)	*p*	Effect estimates (95% CIs)	*p*
Systolic blood pressure, mmHg	4.17 (2.85, 5.49)	<0.0001	0.84 (-0.30, 1.97)	0.1483	1.88 (1.17, 2.59)	<0.0001
Diastolic blood pressure, mmHg	2.33 (1.62, 3.04)	<0.0001	2.50 (1.89, 3.10)	<0.0001	2.61 (2.23, 2.99)	<0.0001
BMI, kg/m^2 *	0.32 (0.21, 0.43)	<0.0001	0.30 (0.20, 0.40)	<0.0001	0.07 (0.01, 0.13)	0.0278
Waist circumference, mm	-4.80 (-8.85, -0.74)	0.0204	-5.81 (-9.28, -2.34)	0.001	-4.89 (-7.07, -2.72)	<0.0001

BMI, body mass index; CI, confidence interval.

The changes in air pollutants and the risk factors were calculated as the differences between the levels at the 2013-14 and 2020-21 resurveys.

The covariates adjusted in the models were the same as those in models of Figure 4.

* Body mass index was not adjusted for.

Table S13. Associations between changes in pollutants (per 10 μg/m^3) and the predicted CVD risks and intermediate risk factors for CVD in the two-pollutant model

	Adjusted pollutant†	$PM_{2.5}$		PM_{10}		O_3	
		Effect estimates (95% CIs)	*p*	Effect estimates (95% CIs)	*p*	Effect estimates (95% CIs)	*p*
Systolic blood pressure, mmHg	O_3	3.93 (2.58, 5.28)	<0.0001	1.10 (-0.19, 2.38)	0.094		
	$PM_{2.5}$					0.19 (-0.64, 1.02)	0.6496
	PM_{10}					-1.11 (-2.19, -0.04)	0.0428
Diastolic blood pressure, mmHg	O_3	2.40 (1.68, 3.13)	<0.0001	1.98 (1.29, 2.67)	<0.0001		
	$PM_{2.5}$					1.88 (1.43, 2.32)	<0.0001
	PM_{10}					0.97 (0.40, 1.55)	0.001
BMI, kg/m^2 *	O_3	0.32 (0.21, 0.44)	<0.0001	0.33 (0.22, 0.44)	<0.0001		
	$PM_{2.5}$					0.10 (0.03, 0.17)	0.004
	PM_{10}					-0.05 (-0.14, 0.04)	0.2887
Waist circumference, mm	O_3	-4.60 (-8.74, -0.46)	0.0293	-3.57 (-7.51, 0.36)	0.0751		
	$PM_{2.5}$					-2.14 (-4.68, 0.40)	0.0987
	PM_{10}					-3.10 (-6.40, 0.20)	0.0657

BMI, body mass index; CI, confidence interval.

The changes in air pollutants and the risk factors were calculated as the differences between the levels at the 2013-14 and 2020-21 resurveys.

Except for the additional adjusted pollutants, the other covariates adjusted in the models were the same as those in models of Figure 4.

* Body mass index was not adjusted for.

† For each additionally adjusted pollutant, the change in pollutant levels between the two resurveys and the levels at the 2013-14 resurvey were included in the model. $PM_{2.5}$ and PM_{10} were not mutually adjusted due to high correlation (Pearson r^2=0.97).

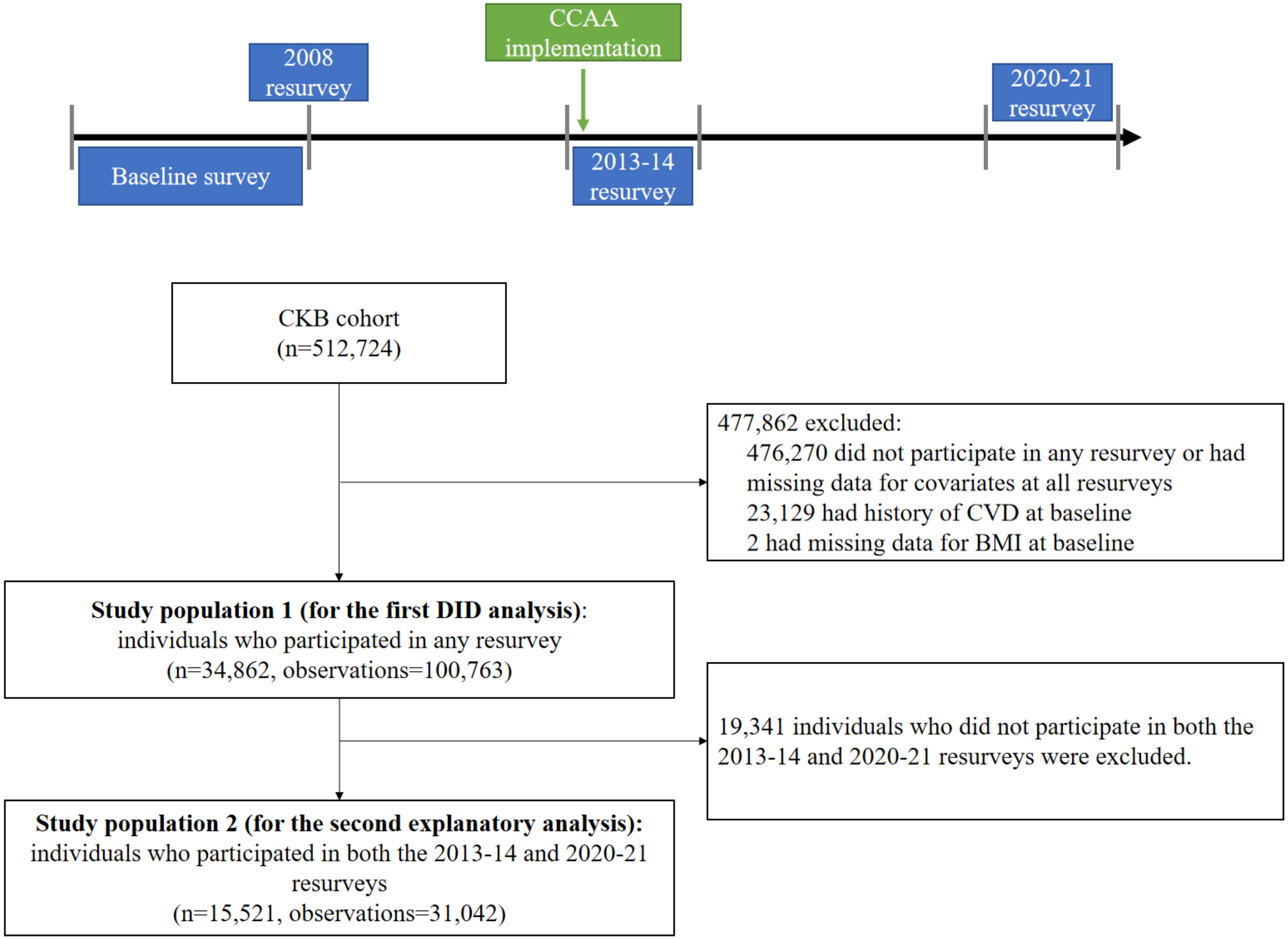

Figure S1. Study design and participants selection

CCAA, China's Clean Air Act; CKB, China Kadoorie Biobank; CVD, cardiovascular disease; DID, difference-in-difference.

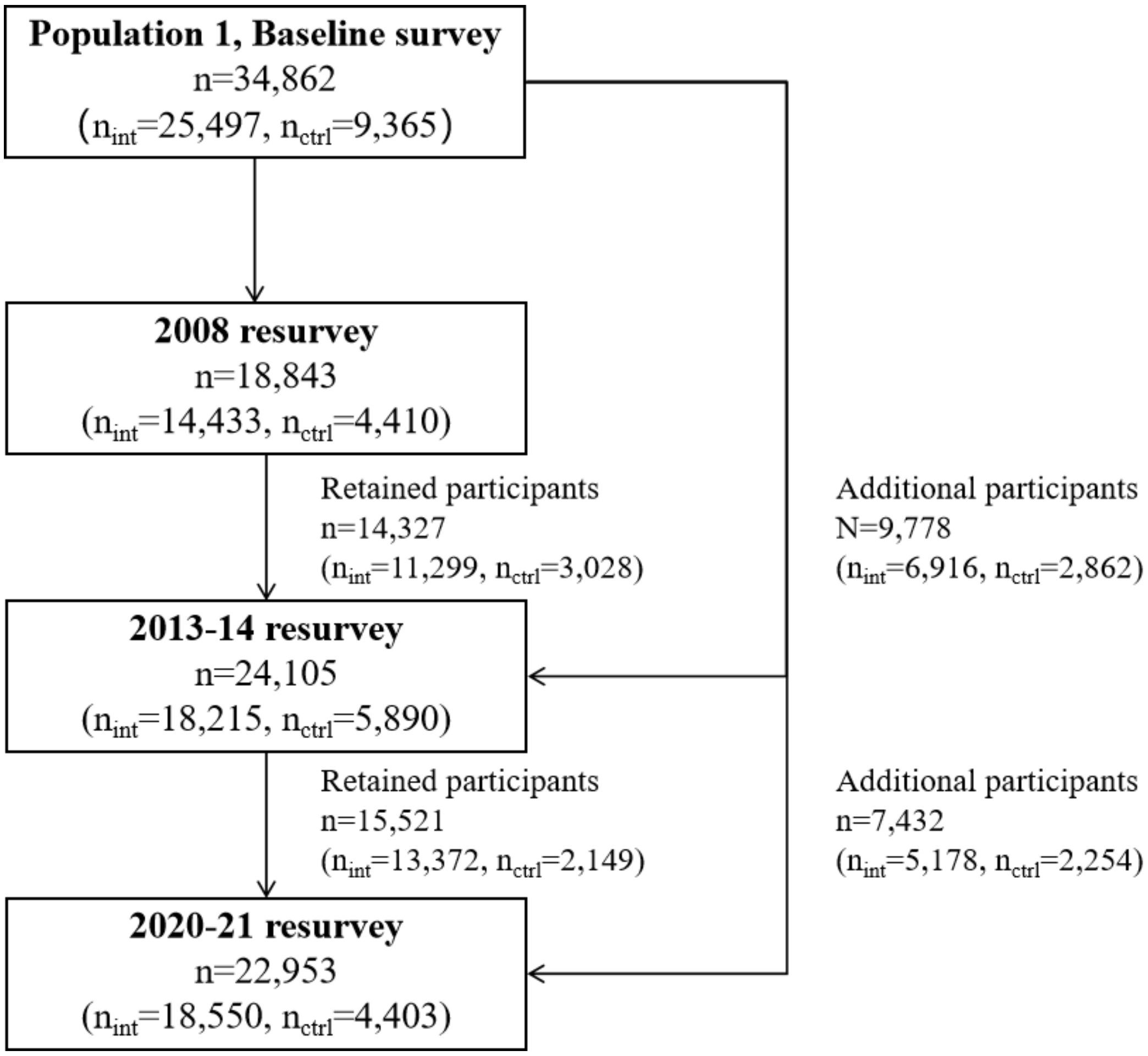

Figure S2. Participants in different survey wave

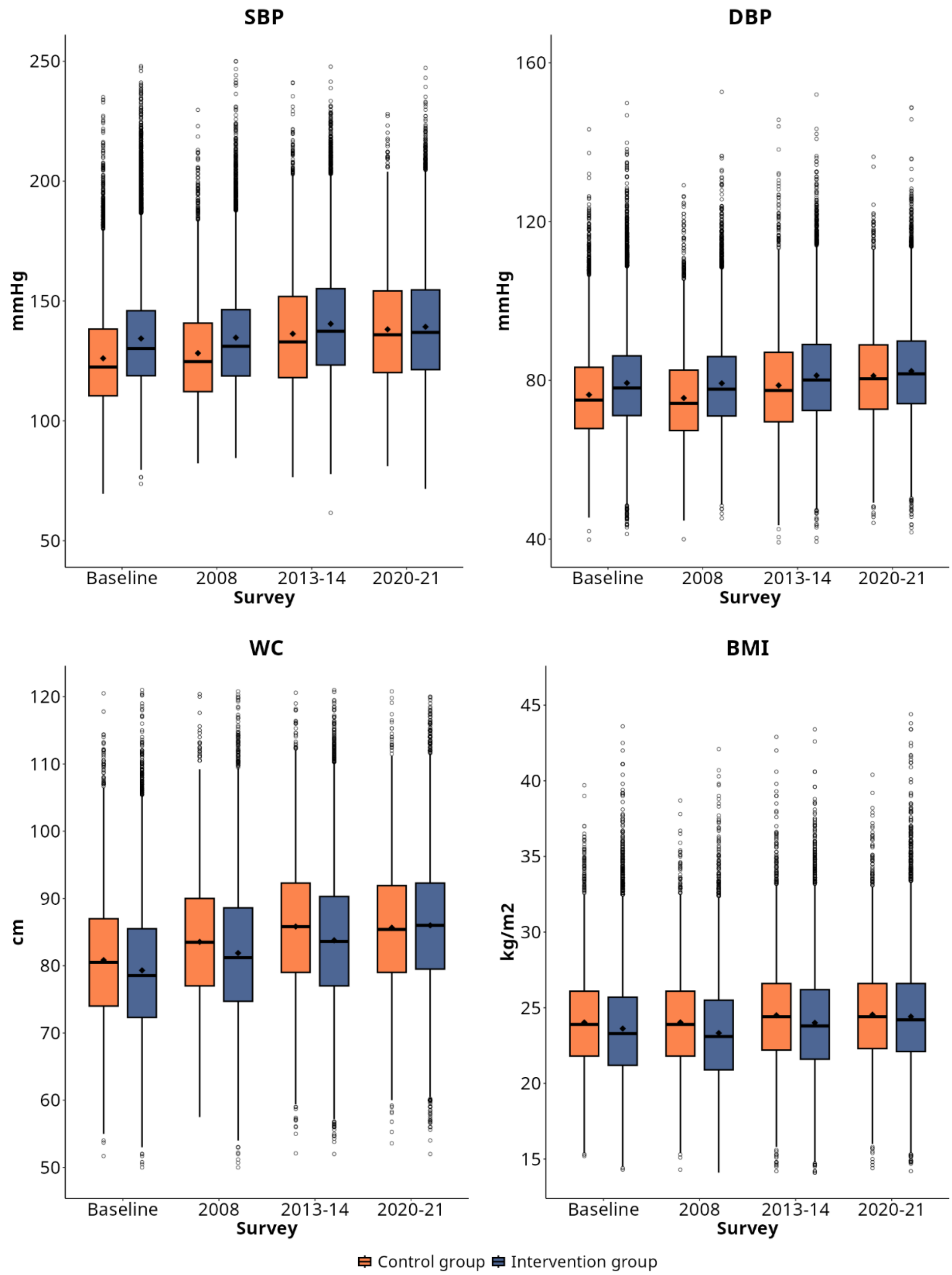

Figure S3. Distribution of intermediate risk factors in each survey

SBP, systolic blood pressure; DBP, diastolic blood pressure; WC, waist circumference; BMI, body mass index.

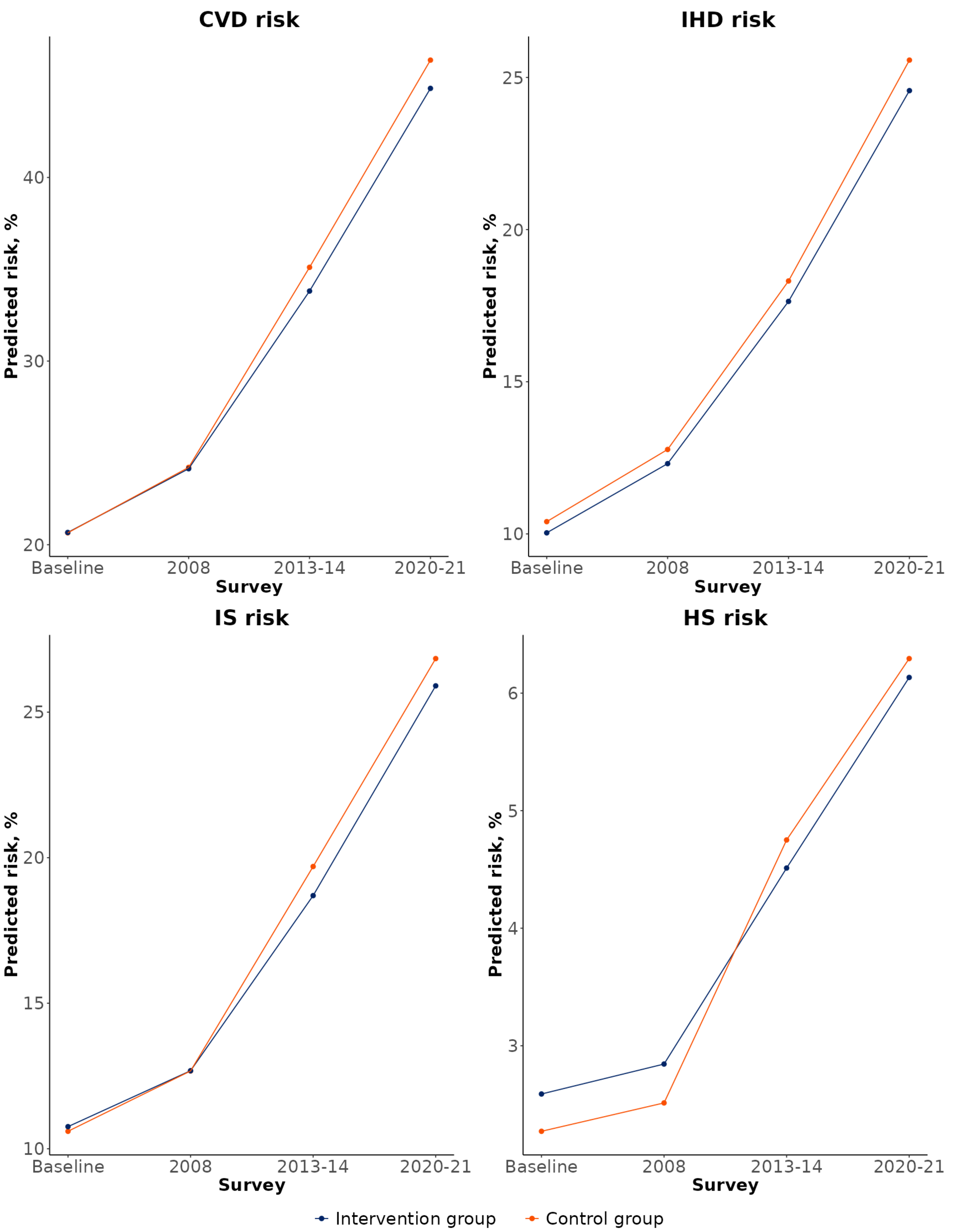

Figure S4. Temporal change of predicted CVD risks in the intervention and control groups
CVD, cardiovascular disease; IHD, ischemic heart disease; IS, ischemic stroke; HS, hemorrhagic stroke**.** Means of predicted CVD risks are shown.

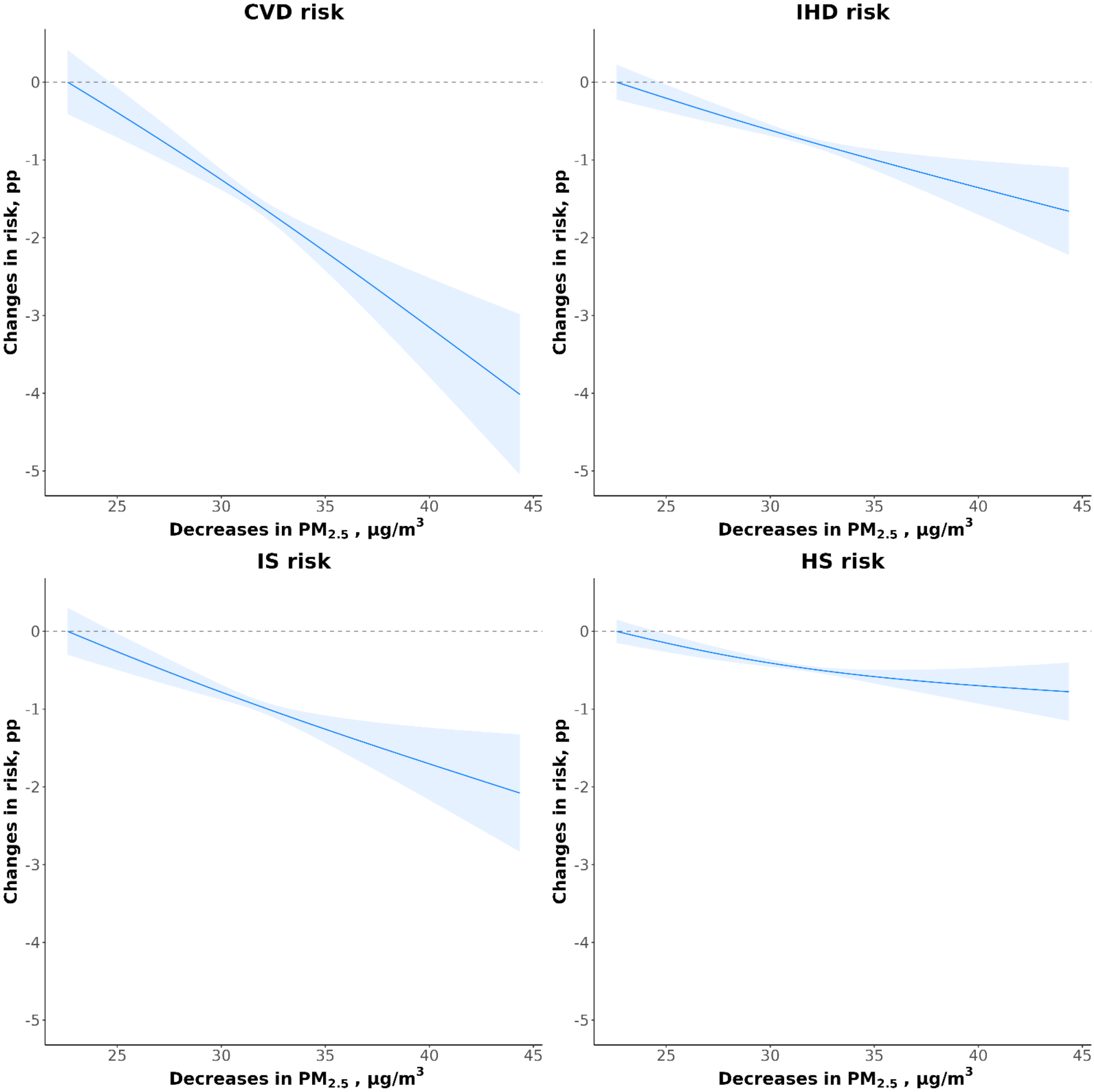

Figure S5. Concentration-response relationship between decreases in $PM_{2.5}$ and the predicted CVD risks

CVD, cardiovascular disease; IHD, ischemic heart disease; IS, ischemic stroke; HS, hemorrhagic stroke; pp, percentage point.

The changes in air pollutants and the predicted outcome risks were calculated as the differences between the levels at the 2013-14 and 2020-21 resurveys.

The covariates adjusted in the models were the same as those in the models of Figure 4.

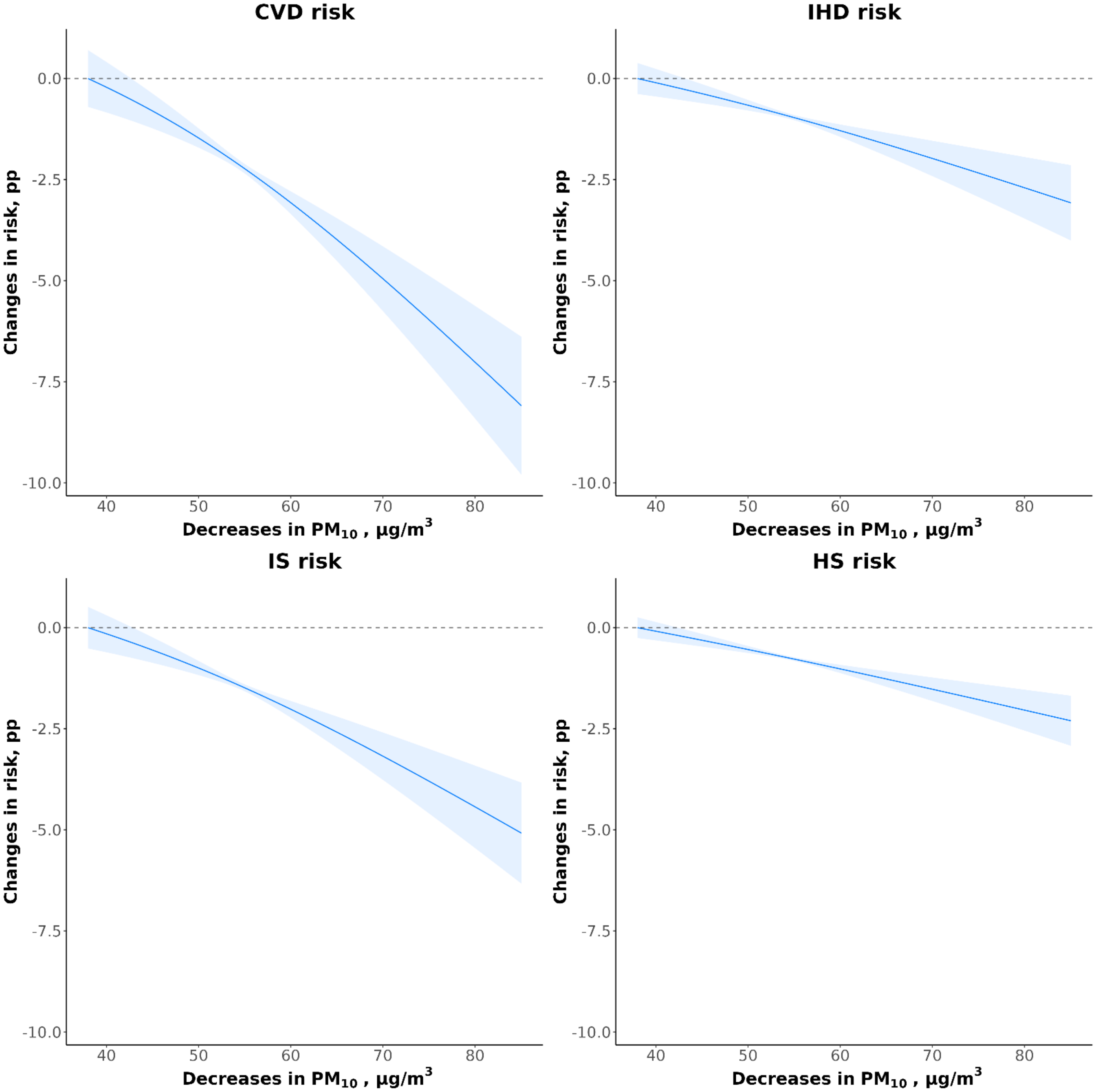

Figure S6. Concentration-response relationship between decreases in PM10 and the predicted CVD risks

CVD, cardiovascular disease; IHD, ischemic heart disease; IS, ischemic stroke; HS, hemorrhagic stroke; β, effect estimates and 95% confidence interval; pp, percentage point.

The changes in air pollutants and the predicted outcome risks were calculated as the differences between the levels at the 2013-14 and 2020-21 resurveys.

The covariates adjusted in the models were the same as those in the models of Figure 4.

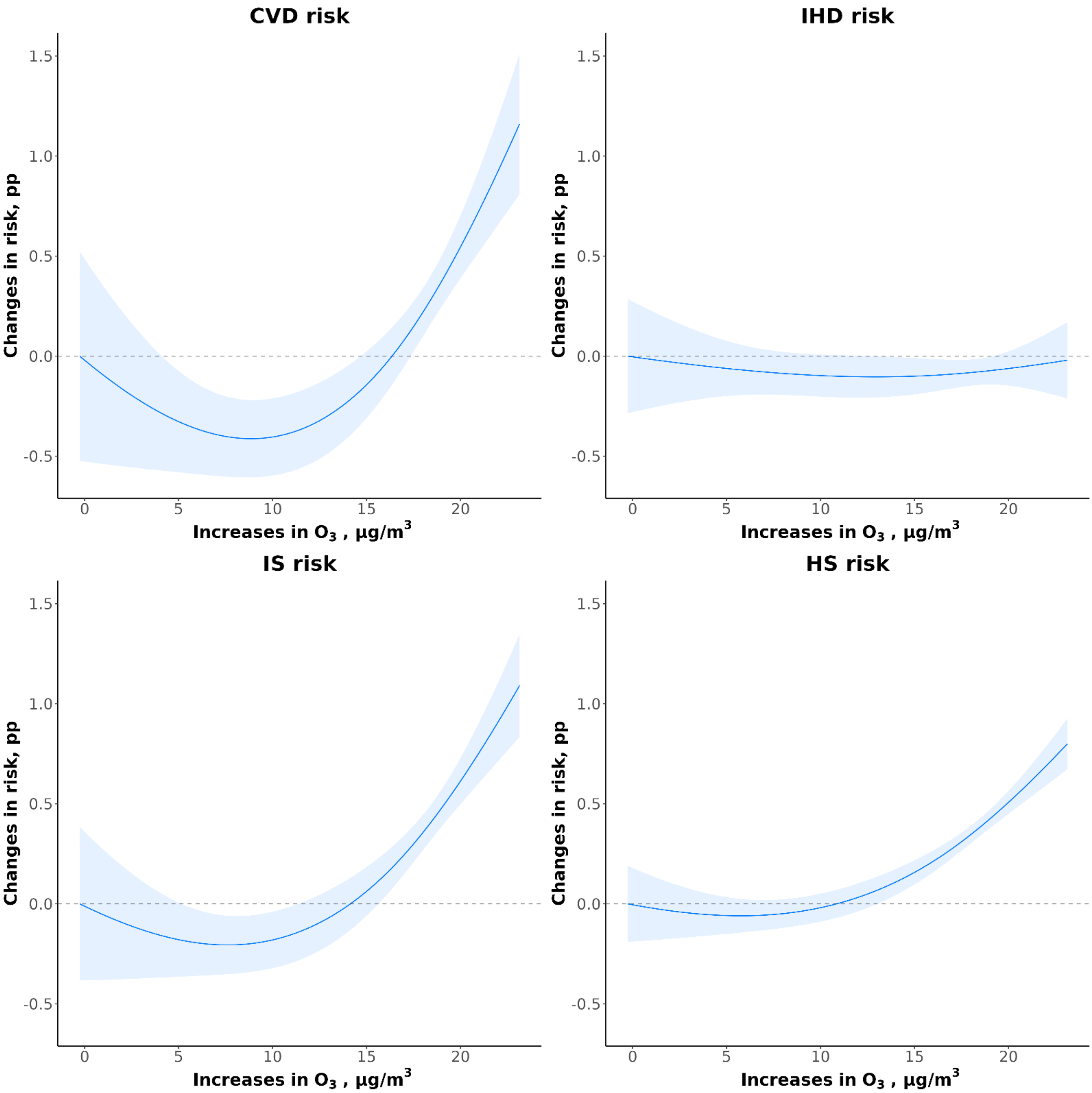

Figure S7. Concentration-response relationship between increases in O_3 and the predicted CVD risks

CVD, cardiovascular disease; IHD, ischemic heart disease; IS, ischemic stroke; HS, hemorrhagic stroke; β, effect estimates and 95% confidence interval; pp, percentage point.

The changes in air pollutants and the predicted outcome risks were calculated as the differences between the levels at the 2013-14 and 2020-21 resurveys.

The covariates adjusted in the models were the same as those in the models of Figure 4.

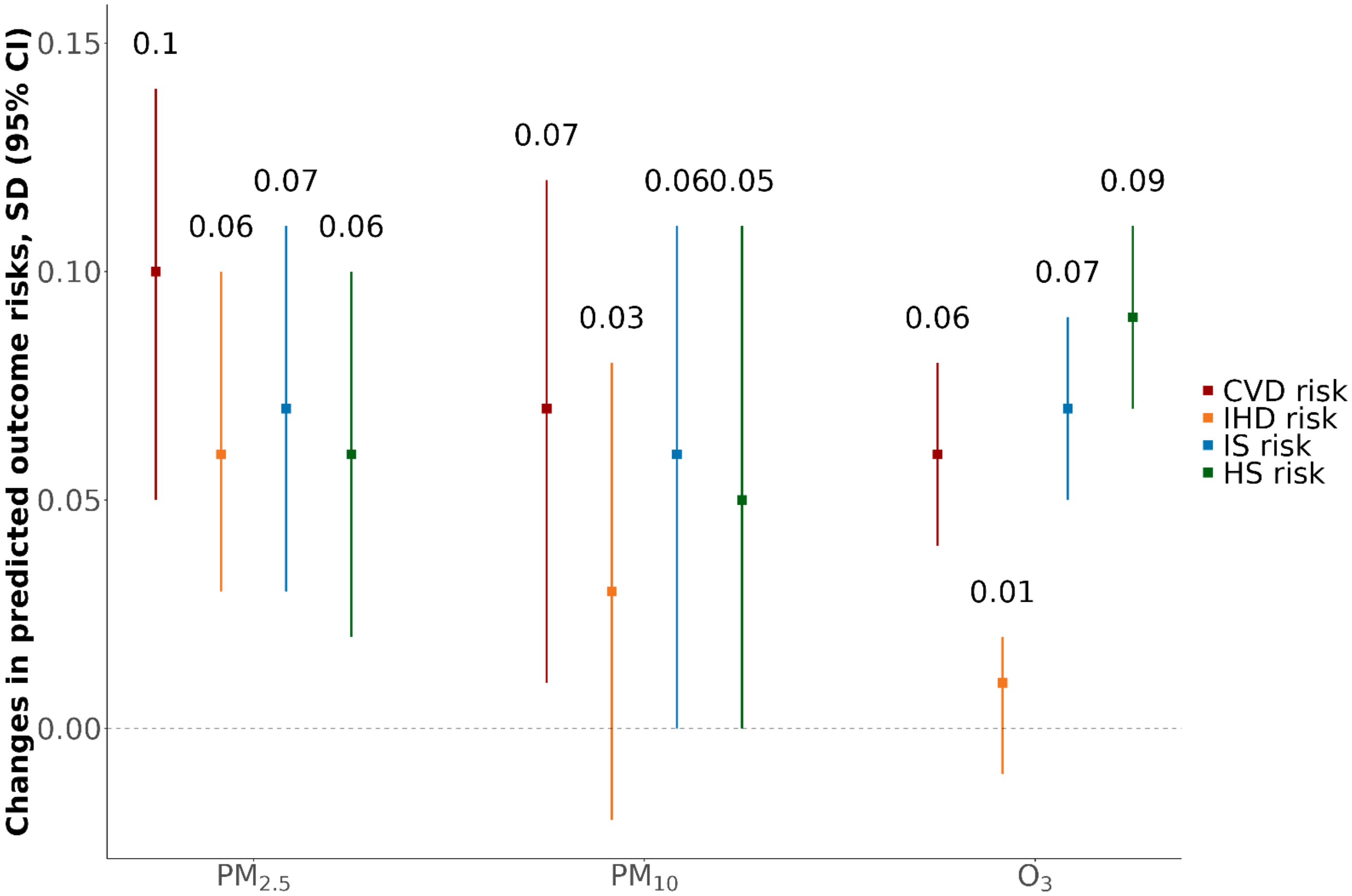

Figure S8. Associations between changes in pollutants (per 10 μg/m³) and per standard deviation change of the predicted cardiovascular disease risks

CVD, cardiovascular disease; IHD, ischemic heart disease; IS, ischemic stroke; HS, hemorrhagic stroke; SD, standard deviation; CI, confidence interval.

The changes in air pollutants and the predicted outcome risks were calculated as the differences between the levels at the 2013-14 and 2020-21 resurveys.

The covariates adjusted in the models were the same as those in the models of Figure 4.

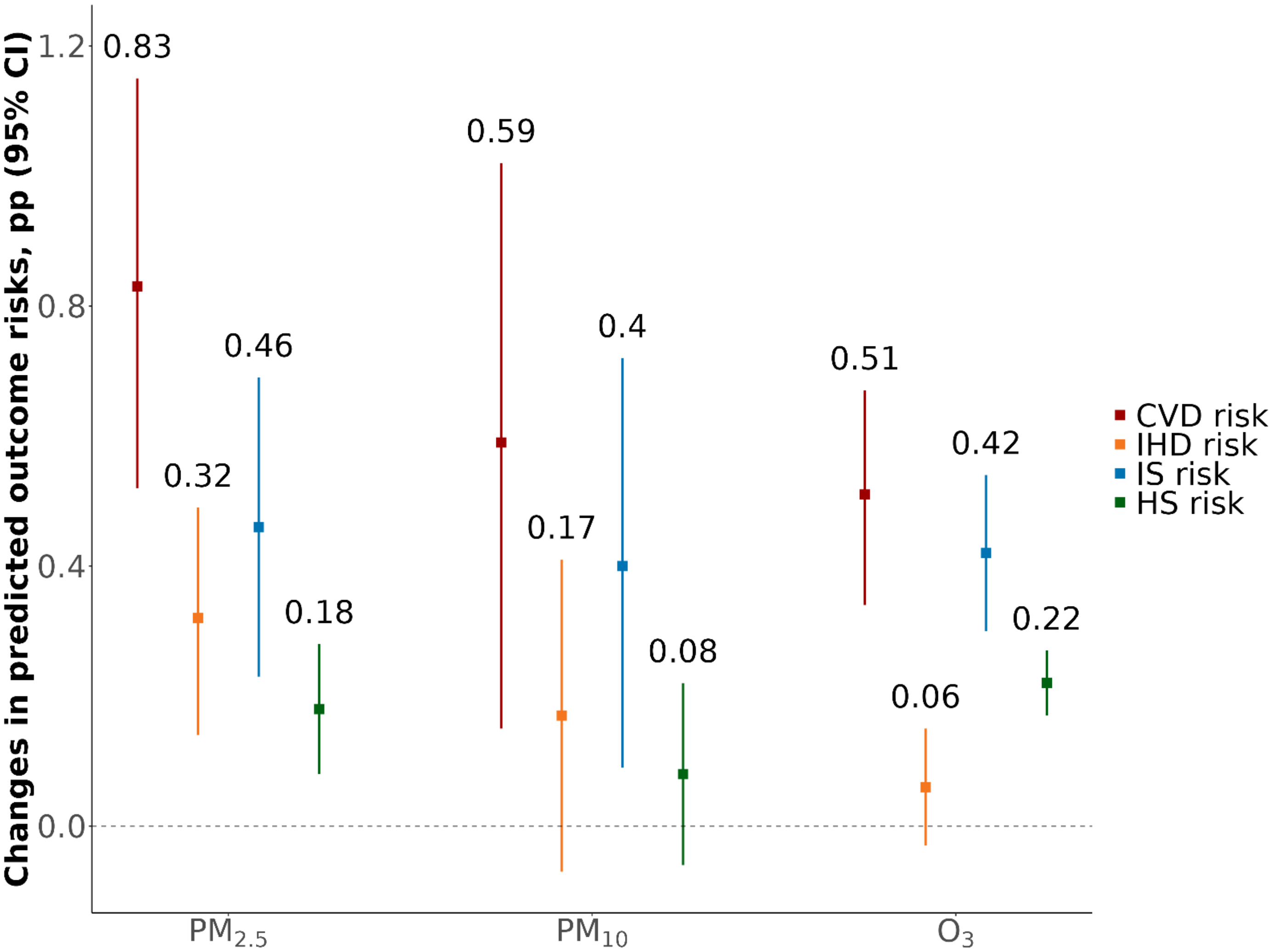

Figure S9. Associations between relative changes in pollutants (per 10% changes) and the predicted cardiovascular disease risks

CVD, cardiovascular disease; IHD, ischemic heart disease; IS, ischemic stroke; HS, hemorrhagic stroke; pp, percentage point; CI, confidence interval.

The relative changes in pollutant levels were calculated by dividing the differences between the levels at the 2013-14 and 2020-21 resurveys by the 2013-14 resurvey levels. The changes in predicted outcome risks were calculated as the differences between the levels at the 2013-14 and 2020-21 resurveys.

Except for not adjusting for pollutant levels at the 2013-2014 resurvey, the other covariates adjusted in the models were the same as those in the models of Figure 4.

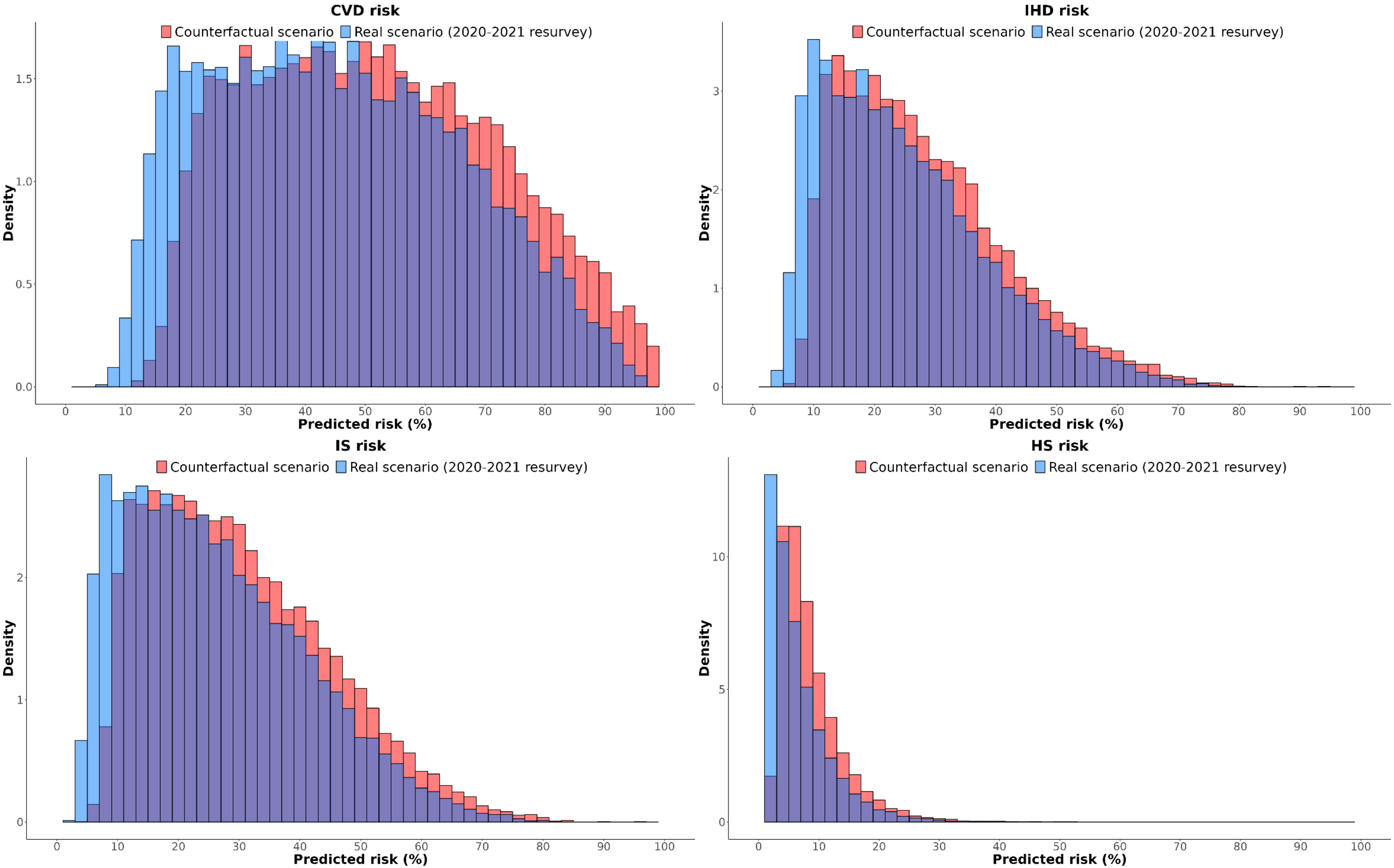
CVD risk
Counterfactual scenario
Real scenario (2020-2021 resurvey)
Density
Predicted risk (%)
IHD risk
Counterfactual scenario
Real scenario (2020-2021 resurvey)
Density
Predicted risk (%)
IS risk
Counterfactual scenario
Real scenario (2020-2021 resurvey)
Density
Predicted risk (%)
HS risk
Counterfactual scenario
Real scenario (2020-2021 resurvey)
Density
Predicted risk (%)

Figure S10. Predicted outcome risks in the real scenario (2020-2021 resurvey) and counterfactual scenario in the absence of changes in $PM_{2.5}$, PM_{10}, and O_3

CVD, cardiovascular disease; IHD, ischemic heart disease; IS, ischemic stroke; HS, hemorrhagic stroke.
